# Global practices, geographic variation, and determinants of child feces disposal in 42 low- and middle-income countries: An analysis of standardized cross-sectional national surveys from 2016 – 2020

**DOI:** 10.1016/j.ijheh.2022.114024

**Published:** 2022-08

**Authors:** Stephen G. Mugel, Thomas F. Clasen, Valerie Bauza

**Affiliations:** Gangarosa Department of Environmental Health, Rollins School of Public Health, Emory University, 1518 Clifton Rd. NE, Atlanta, GA, 30322, United States

**Keywords:** Child feces management, Child feces disposal, Sanitation, WaSH, DAL, Disposal of child's feces in any type of latrine, DIL, Disposal of child's feces in an improved latrine, CFD, Child feces disposal

## Abstract

**Background:**

Despite considerable progress improving water and sanitation access globally, unsafe child feces disposal remains common in many low- and middle-income countries (LMICs), posing an important health risk. The present study characterizes the current prevalence of child feces disposal practices and child latrine use across low- and middle-income countries and investigates determinants associated with appropriate disposal practices.

**Methods:**

Data for children ranging from 0 through 4 years of age were analyzed from standardized and nationally-representative surveys of 42 LMICs collected from 2016 to 2020 to assess child feces disposal practices. We report child feces disposal in three categories: disposal in any type of latrine, disposal in an improved latrine, and disposal through means other than in a latrine. Survey weighted multiple Poisson regression models were used to explore factors associated with these practices.

**Results:**

Data on 403,036 children (weighted N = 191 million) demonstrated that a minority (40.3%) of children have their feces disposed of in a latrine of any kind, and just 29% have feces disposed of in an improved latrine. Prevalence varied considerably by country and region. In adjusted analyses, both child feces disposal in any latrine and disposal in an improved latrine increased with child age, higher intra-country relative wealth, and urban living, and decreased with breastfeeding and shared sanitation facilities. Disposal in improved latrines additionally increased with access to higher levels of service for drinking water and higher mother's education. Nevertheless, the role of facility access alone was insufficient, as only about half of children with household access to any latrine or improved latrines had their feces disposed of in these facilities. Child latrine use among households with latrine access was also low and highly variable across countries.

**Conclusions:**

Children's feces in LMICs are infrequently disposed of in any latrine type, and even less frequently in improved latrines. In order to minimize health risks in LMICs, increased effort must be undertaken not just to increase sanitation coverage but to address these common barriers to safe child feces disposal and child latrine use.

## Highlights

• Global child feces disposal in latrines is low and variable between countries.
• Latrine access is insufficient to explain low use for child feces management.
• Household relative wealth is associated with increased disposal in latrines.
• Child latrine use increases with age but remains low and varied between countries.

## 1 Introduction

Poor access to sanitation in low- and middle-income countries (LMICs) is associated with a large burden of disease, including diarrheal disease, soil-transmitted helminth infections, schistosomiasis, trachoma, and child undernutrition (Freeman et al., 2017; Prüss-Ustün et al., 2019). However, even households with access to sanitation facilities often do not dispose of their young children's feces into their latrine when the child defecates elsewhere (Bauza et al., 2019b; Bauza and Guest, 2017; Majorin et al., 2017). Inadequate disposal of child feces presents a significant source of exposure and associated health risks. Young children often have underdeveloped immune systems and more frequent diarrheal disease which may lead to higher pathogen loads in their feces (Feachem et al., 1984; Walker, 2018). It is also common for young children to defecate inside or close to households, with past research identifying fecal contamination from young children's feces to be more common inside households than contamination from older children or adult's feces (Bauza et al., 2019a). As a result, susceptible children within the same or nearby households may be more likely to be exposed to feces from other young children, as children spend much time on the ground engaging in exploratory behaviors that include mouthing of hands, objects, and soil (Bauza et al., 2018; Kwong et al., 2016; Moya et al., 2004; Ngure et al., 2013). Consistent with this potential exposure route, past research has found unsafe child feces disposal to be associated with diarrhea (Majorin et al., 2019b), soil-transmitted helminth infection (Roy et al., 2011), environment enteric dysfunction (George et al., 2016), and stunting (Bauza and Guest, 2017) in children.

For children's feces to be safely managed, all points of potential exposure to pathogens from the feces must be blocked, including at the defecation and feces disposal sites as well as the material used for feces handling, child and caregiver hands, and the site and any materials used for anal cleansing (Bauza et al., 2020; Majorin et al., 2017). Despite the noted importance of many of these exposure points in the World Health Organization's *Guidelines on Sanitation and Health* (WHO 2018), international monitoring focuses exclusively on the disposal site of child feces. Historically, the WHO/UNICEF Joint Monitoring Program on Water, Sanitation and Hygiene (JMP) has defined “safe” child feces disposal as a child using a toilet facility or the child's feces being put into a latrine or buried, with the type of toilet facility not being considered. However, burial was later recommended against as a safe method of disposal following an expert consultation due in part to potential for contamination from buried feces to spread from animals or rain (Bain and Luyendijk, 2015). More recently, the JMP has updated what they consider to be “appropriate” disposal of child feces to include a child using an improved latrine or their feces being disposed of in an improved latrine or disposed with solid waste if that solid waste is stored, collected, and disposed of in a sanitary manner (UNICEF/WHO 2018).

Although some past studies have measured the scope or determinants of safe disposal of child feces, these studies are usually on a local or regional level within a specific country (Azage and Haile, 2015; Bauza et al., 2019b; Majorin et al., 2017; Sahiledengle, 2020) or region (Seidu et al., 2021). Moreover, no large multi-country studies have documented the scope and variation of child latrine use in LMICs, a behavior which also eliminates other sources of exposure that could be associated with defecation outside the latrine such as feces handling or contamination of the site of defecation. Overall, there is still limited evidence on the scope and determinants of safe CFD and child latrine use in LMICs on a global level based on recent data.

The objective of this research is to characterize the prevalence of different child feces disposal practices in LMICs and assess the personal, household, environmental, and community factors that are associated with safe disposal in a latrine. A secondary objective is to characterize the scope and variation of child latrine use across child age and countries. The knowledge from this study can help identify the scope and enabling factors to safe child feces disposal practices in LMICs.

## 2 Methods

### 2.1 Data sources

We analyzed data collected from households with young children from nationally representative surveys conducted within the past five years (2016–2020) in 42 LMICs within Sub-Saharan Africa, South Asia, East Asia and Pacific, Latin America and Caribbean, and Middle East and North Africa regions. This includes data from both Demographic and Health Survey (DHS) and Multiple Indicator Cluster Survey (MICS) datasets. All country surveys from LMICs in this time period which asked questions on child feces disposal (CFD) were included*.*

The DHS survey is administered by USAID in LMICs to women aged 15–49 in households selected by a stratified random sample designed to be representative of the population of the country and asks questions regarding household characteristics and women and children's health (Corsi et al., 2012). The DHS survey administers a CFD question regarding only the youngest child under 2 years old (except Afghanistan, India, and Myanmar, where the question is asked of the youngest child under 5 years old). The question on CFD is posed as: “The last time [name of child] passed stools, what was done to dispose of the stools?”(UNICEF/WHO, 2018). Possible responses include: ‘child used toilet/latrine,’ ‘put/rinsed into toilet or latrine,’ ‘put/rinsed into drain or ditch,’ ‘thrown into garbage (solid waste),’ ‘buried,’ ‘left in the open,’ or ‘other.’ The MICS survey is similarly designed and nationally representative, and is administered by UNICEF in sections to the head of household and women aged 15–49. While many questions cover all children up to 5 years old, the CFD question is posed regarding only children under 3 years old (Khan and Hancioglu, 2019). The question is posed in the same way as DHS.

### 2.2 Child feces disposal practice definitions

The Joint Monitoring Program on Water, Sanitation and Hygiene (JMP) guidelines categorizes sanitation facilities as ‘improved’ or ‘unimproved’. ‘Improved latrines’ are “those designed to hygienically separate excreta from human contact, and include: flush/pour flush toilets connected to piped sewer systems, septic tanks or pit latrines; pit latrines with slabs (including ventilated pit latrines), and composting toilets”; unimproved latrines are pit latrines without a slab or platform, hanging latrines or bucket latrines (UNICEF/WHO, 2021). The JMP now employs additional rungs in its ‘sanitation ladder’ with ‘open defecation’ at the bottom, followed by ‘unimproved latrines,’ and improved latrines further categorized depending on whether they are shared (‘limited’), unshared (‘basic‘) or unshared with fecal waste safely disposed in-situ or treated offsite (‘safely managed’). Data on fecal waste management classifications of improved latrines are unavailable for DHS datasets for the years covered by this analysis, so ‘basic’ and ‘safely managed’ are combined into a single category.

**Fig. 1:**
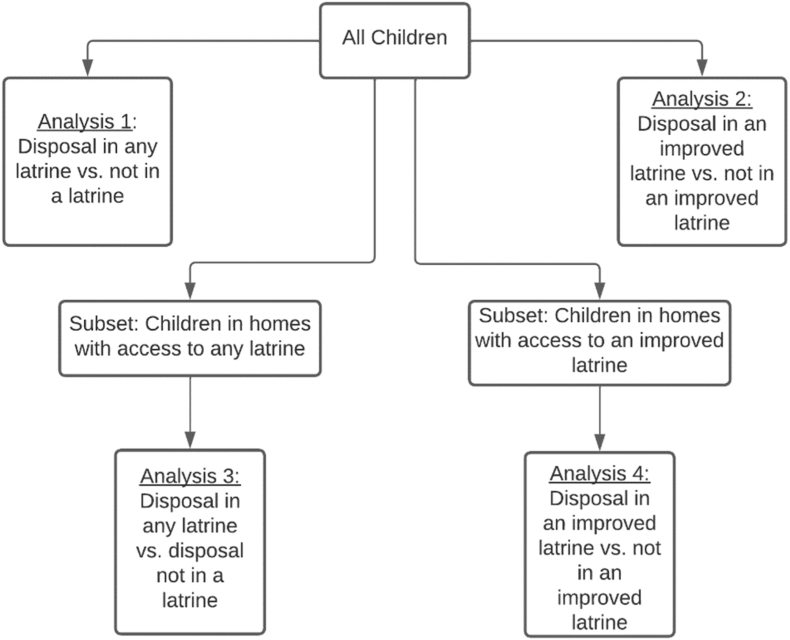
Diagram of each binary analysis to investigate child feces disposal practices. Analyses include (1) whether disposal is in any latrine (DAL), (2) whether disposal is in an improved latrine (DIL), (3) DAL conditional on access to any latrine and (4) DIL conditional on access to an improved latrine.

Child feces disposal was analyzed in two parallel ways, each as binary outcomes (Fig. 1). First, binary outcomes of disposal in any type of latrine (DAL = ‘yes; ’ defined by responses of ‘used latrine,’ or ‘put/rinsed into latrine’ to the child feces disposal question) were compared against disposal not in a latrine (DAL = ‘no; ’ defined by responses of ‘put/rinsed into a drain or ditch,’ ‘thrown in garbage/solid waste,’ ‘buried,’ ‘left in open/not disposed of,’ or ‘other’ to the child feces disposal question) (Analysis 1). As ‘safely managed sanitation’ requires as a starting point that feces be contained in an ‘improved’ latrine, the second analysis compared children whose feces are disposed of in an improved latrine (DIL = ‘yes’) against children whose feces were not disposal of in an improved latrine (DIL = ‘no’) (Analysis 2). For this purpose, disposal in improved latrines was defined by the respondent indicating that the last time the child defecated the child ‘used latrine’ or their feces were ‘put/rinsed into latrine’ *and* the respondent indicated that the household uses a latrine that met the ‘safely managed,’ ‘basic,’ or ‘limited’ definitions of ‘improved’ sanitation. Disposal not in improved latrines was defined by respondents indicating that the last time the child defecated the feces were ‘put/rinsed into a drain or ditch,’ ‘thrown in garbage/solid waste,’ ‘buried,’ ‘left in open/not disposed of,’ or ‘other,’ *or* if they used a latrine but the latrine in the household only met the JMP definition for an ‘unimproved’ latrine. As the included surveys did not allow us to verify if solid waste was stored, collected, and disposed of in a sanitary manner, we have classified the disposal of child feces with solid waste as inadequate disposal for this analysis, similar to other disposal options that were not in a latrine. Additionally, as safe disposal of child feces is a behavioral practice and is not simply a function of having access to a latrine, additional analyses were conducted to explore the extent to which the subset of households with access to any latrine (Analysis 3) and improved latrines (Analysis 4) reported using the same for the disposal of child feces.

### 2.3 Predictor variable definitions

The ages of children and mothers were recorded in months and converted to decimal years. The education level of mothers was grouped into three categories: less than primary, primary, and secondary or higher education levels. Whether or not the child is currently being breastfed was recorded. Intra-country wealth quintiles at the household level were calculated by DHS and MICS methodology from all households surveyed (i.e. not just from those included in this dataset). Urbanicity, defined in the original datasets as ‘urban’ or ‘rural,’ or ‘camp,’ was re-grouped to include the camp with rural designation due to sparse data (n = 446 and n = 1,084 children from Suriname and the State of Palestine, respectively). The number of other children under 5 years and total persons in the house was recorded in original datasets, and these variables were grouped as none versus 1 or more additional children (2 or more total children <5 years in the households), and less than five persons versus 6 or more, respectively, based on distributions of the data. Whether or not a latrine is shared amongst multiple families was included as a binary variable.

The quality of drinking water was included based on the JMP ladder grouped into four categories: a group of ‘surface water’ and ‘unimproved’ together (due to sparse surface water data), ‘limited,’ ‘basic,’ and ‘safely managed’ (UNICEF/WHO, 2018). The source of water for sanitation and hygiene was recorded only for MICS datasets, and therefore not included in the analysis.

### 2.4 Sample weighting

Due to the hierarchical cluster sampling procedures of the DHS and MICS protocols, the samples are weighted to be nationally representative (Corsi et al., 2012). DHS datasets weight data at the level of women respondents, and only ask about CFD for their youngest child under 2 years old (or under 5 years old in select countries). In MICS datasets, data are weighted at the level of the child (under 5 years old) while CFD questions were only asked regarding children 0–2 years old. Therefore, to compare weighted data across countries, weights were first denormalized by multiplying each weight by the UN estimated 2015 population of women aged 15–49 (for DHS) or children under 5 (for MICS), then dividing by the total number of surveyed women for that country (from DHS country-specific final reports), or the number of children (from MICS country-specific final reports) (Corsi et al., 2012). Survey weights were denormalized from each country prior to prevalence calculations, descriptive analyses, and statistical analyses to allow for pooled data interpretation.

### 2.5 Statistical analysis

All analyses were performed in STATA v16 (StataCorp LLC, College Station, TX, USA). Sample weighting and hierarchical cluster sampling was accounted for in our analysis by using the ‘*svyset*’ command. Primary sampling units (PSU) were set by the survey design in each country, and the sampling strata were formed by combining the country, national region, and urban vs. rural designation. Descriptive univariate analyses accounting for survey design and weighting, including prevalence of outcome variables by country and region, were conducted using the denormalized weights.

Multivariate Poisson regression was used to assess potential determinants of child feces disposal practices. Estimates of prevalence ratios (PRs) for variables associated with child feces disposal practices (DAL and DIL, Analyses 1 and 2, respectively) were generated using survey-weighted multiple Poisson regression models using denormalized weights so data were representative and comparable across countries, and using clustering at the sampling strata (primary sampling unit) level. To account for general between-country differences, each country was adjusted for by treating it as a fixed factor in the model (results shown in supplement). To further explore factors associated with DAL and DIL after accounting for sanitation facility access alone, these models were run again after restricting to only observations with household access to any sanitation facility (for DAL as the response, Analysis 3) and access to an improved sanitation facility (for DIL as the response, Analysis 4).

### 2.6 Sensitivity analyses

To explore how robust this analysis was, the model was run using a number of permutations. For all analyses except one, the denormalized weighting and survey clustering scheme of the main analysis was used. India accounted for roughly 40% of the observations, therefore we ran the analyses dropping India from the dataset to test if the results were overly influenced by this single country (New n = 217,607, 185,429 observations deleted). We also tested an alternative weighting scheme in which every country was given equal weight.

To standardize the population age between all datasets, we performed an analysis with all children older than 2-years-old dropped (thereby dropping observations of children 2 years old from MICS datasets and dropping children 2 and older for select DHS datasets which included information on children less than 5 years old; new n = 266,817, and 136,760 observations deleted). We also conducted an analysis in which we additionally standardized the household level sampling methodology between DHS and MICS datasets by including from MICS only the youngest child of each mother under 2 years old (new n = 357,865, and 47,204 observations deleted). We also accounted for possible correlation among observations from within the same household (MICS only) by adding an additional clustering term to the model at the household level.

## 3 Results

### 3.1 Sample demographic characteristics

The final sample for analysis included 42 countries and 403,036 children (N = 389,611 with sufficient information on sanitation included necessary for DIL definition). These were comprised of 24 DHS datasets (n = 297,741 children (73.78%)), and 18 MICS datasets (n = 105,836 children (26.22%)). Following denormalization, the weighted data represent more than 191 million observations, and are intended to be a representative sample of the more than 229 million children living in the LMICs included in this dataset (Table 1).

**Table 1:** Description of the data sources used in the analysis. Sample sizes (unweighted and *denormalized* weights), the estimated total population of children in the country from UN projections of 2015^a^, and the weighted composition of each country to the region and the overall dataset are displayed.

Region	Country	Survey Type	Year Survey Completed	N children surveyed	Weighted N children	2015 Child Population in Age Range^a^	Age Range (Years)	Weighted Percent by Region	Weighted Percent Total Dataset
East Asia and Pacific	Indonesia	DHS	2017	6,658	8,957,166	10,164,628	0–1	52.7	4.68
	Mongolia	MICS	2018	3,420	199,898	226,958	0–2	1.18	0.1
	Myanmar	DHS	2016	3,767	3,984,821	4,574,474	0–4	23.44	2.08
	Philippines	DHS	2017	3,766	3,795,277	4,663,949	0–1	22.33	1.99
	Timor-Leste	DHS	2016	2,692	60,685	64,277	0–1	0.36	0.03
	Total			**20,303**	**16,997,847**				**8.89**
Latin America and Caribbean	Costa Rica	MICS	2018	2,057	207,164	214,947	0–2	20.34	0.11
	Cuba	MICS	2019	2,870	345,900	387,010	0–2	33.97	0.18
	Haiti	DHS	2017	2,352	437,216	514,473	0–1	42.94	0.23
	Suriname	MICS	2018	2,371	27,987	31,972	0–2	2.75	0.01
	Total			**9,650**	**1,018,267**				**0.53**
Middle East and North Africa	Algeria	MICS	2019	8,623	2,645,683	2,849,428	0–2	40.29	1.38
	Iraq	MICS	2018	9,572	2,959,383	3,253,717	0–2	45.06	1.55
	State of Palestine	MICS	2020	3,906	412,594	416,280	0–2	6.28	0.22
	Tunisia	MICS	2018	1,883	549,640	633,660	0–2	8.37	0.29
	Total			23,984	6,567,300				3.43
South Asia	Afghanistan	DHS	2016	19,207	4,997,566	5,500,914	0–4	4.41	2.61
	Bangladesh	MICS	2019	13,570	8,069,534	8,811,102	0–2	7.12	4.22
	India	DHS	2016	185,429	86,410,488	118,983,308	0–4	76.23	45.2
	Maldives	DHS	2017	1,136	14,969	14,485	0–1	0.01	0.01
	Nepal	MICS	2016	3,719	1,554,700	1,682,271	0–2	1.37	0.81
	Pakistan	DHS	2018	4,477	12,310,060	10,710,158	0–1	10.86	6.44
	Total			**227,538**	**113,357,317**				**59.29**
Sub-Saharan Africa	Angola	DHS	2016	5629	2,303,263	2,166,002	0–1	4.33	1.2
	Benin	DHS	2018	5,265	821,713	725,532	0–1	1.54	0.43
	Burundi	DHS	2017	5,094	720,716	780,147	0–1	1.35	8.89
	Cameroon	DHS	2018	3,562	1,524,666	1,582,119	0–1	2.86	0.8
	Central African Republic	MICS	2019	4,992	402,799	444,428	0–2	0.76	0.21
	Chad	MICS	2019	11,824	1,452,347	1,632,666	0–2	2.73	0.76
	Democratic Republic of the Congo	MICS	2018	12,393	8,122,085	8,778,256	0–2	15.25	4.25
	Ethiopia	DHS	2016	3,914	6,365,234	6,451,717	0–1	11.95	3.33
	Ghana	MICS	2018	4,989	2,194,674	2,428,707	0–2	4.12	1.15
	Guinea	DHS	2018	2,825	951,202	793,358	0–1	1.79	0.5
	Lesotho	MICS	2018	1,652	112,509	152,151	0–2	0.21	0.06
	Madagascar	MICS	2018	7,462	2,091,566	2,263,462	0–2	3.93	1.09
	Malawi	DHS	2016	6,383	1,033,245	1,112,165	0–1	1.94	0.54
	Nigeria	DHS	2018	12,076	12,046,369	13,079,711	0–1	22.62	6.3
	Sao Tome and Principe	MICS	2019	1,051	17,504	18,756	0–2	0.03	0.01
	Senegal	DHS	2018	5,168	1,786,208	1,014,324	0–1	3.35	0.93
	Sierra Leone	DHS	2019	3,637	396,997	449,300	0–1	0.75	0.21
	South Africa	DHS	2016	1,223	2,160,530	2,318,621	0–1	4.06	1.13
	Tanzania	DHS	2016	4,035	3,601,115	3,609,576	0–1	6.76	1.88
	The Gambia	MICS	2018	5,530	200,969	227,019	0–2	0.38	0.11
	Uganda	DHS	2016	5,642	2,666,624	2,917,723	0–1	5.01	1.39
	Zambia	DHS	2019	3,804	1,028,679	1,141,807	0–1	1.93	0.54
	Zimbabwe	MICS	2019	3,411	1,252,703	1,393,489	0–2	2.35	0.66
	Total			**121,561**	**53,253,717**				**27.85**
Grand total				**403,036**	**191,194,448**	**229,179,047**			

a UN data population estimates for 2015 for children aged 0–2 years for MICS countries, for 0–1 years for DHS countries, except Afghanistan, Myanmar, and India, which were from 0 to 4 years.

Overall the total dataset spanned five regions, with the majority of countries from Sub-Saharan Africa (23 out of 42). Among included children, there were slightly more male than female children (47.6% female). The mean age was 1.16 years, and 65.8% of children were currently breastfeeding at the time of the interview. 66.9% of children were from rural areas, with roughly 20% in each of five wealth quintiles. 51.7% of children were in a household with 2 or more children under 5 years old, and 55.7% were in households of 6 or more persons (see Table S1 for summary of weighted descriptive data). Mothers were an average of 27.7 years old, and 51.0% had secondary or higher education, with 20.5% attaining only primary and 28.5% attaining less than primary education. There was considerable variation in these demographic characteristics among countries (see Table S2 for country-specific socio-demographic data).

Sanitation and water access were moderate across the study population. In the overall dataset, 29.3% of children lived in households that practiced open defecation, while 12.9% had access to unimproved sanitation facilities, 13.0% had access to limited sanitation facilities, and 44.8% had access to basic or safely managed sanitation facilities, according to the JMP guidelines. 24.1% of children were from households that shared their sanitation facility. 13.6% of households had access to only surface or unimproved water, 17.8% had access to limited water, 24.7% had access to basic water, and 43.9% had access to safely managed water. There was also considerable variation in water and sanitation among countries (see Table S3 for country-specific data on sanitation and water).

### 3.2 Child feces disposal prevalence and scope

**Fig. 2:**
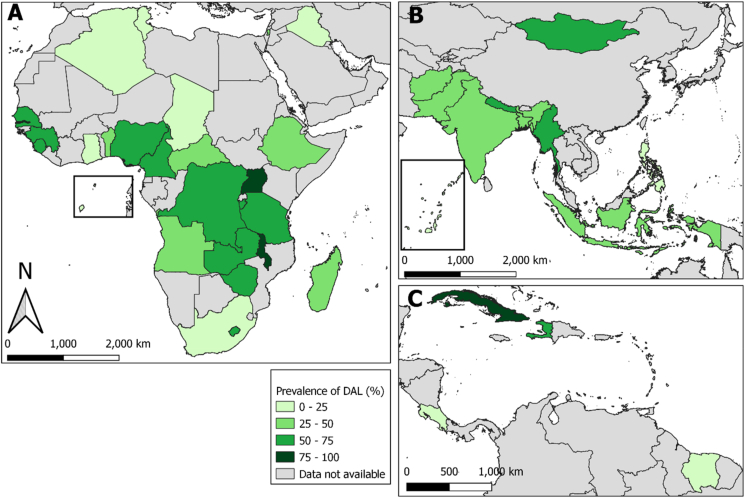
Map of the prevalence of safe disposal of feces in a latrine of any kind (DAL) across LMICs included in the DHS and MICS surveys from 2016 to 2020. Grey countries did not have data included in this analysis, and darker green corresponds to higher prevalence of DAL. (A) depicts the countries included from WHO designated regions of Sub-Saharan Africa and North Africa and the Middle East, including an inset for Sao Tome and Principe. (B) depicts the regions of South Asia and East Asia and the Pacific, with an inset for the most populated islands of the Maldives. (C) depicts the countries included from Latin America and the Caribbean. A map of the prevalence of disposal in improved latrines (DIL) shown in the supplement (Fig. S1). (For interpretation of the references to colour in this figure legend, the reader is referred to the Web version of this article.)

Disposal of feces in a latrine of any kind (DAL) was reported for 40.3% of children, while disposal of feces in a latrine meeting the ‘improved’ standard (DIL) was reported for only 29.0% of children (Table 2). There was wide variation in the prevalence of DAL across countries and regionally, as seen in Fig. 2 (Table 2; DIL shown in Fig. S1). Overall 13.2% of children used a latrine directly, while 27% of children had their feces deposited into a latrine (presumably by a caregiver), and 23.9% of children's feces were left in the open or not disposed of in any manner (Table 2). There was considerable variation among countries in child feces disposal practices.

**Table 2:** Prevalence (shown as percentages) of specific methods of child feces disposal (CFD) using denormalized weights by country, including prevalence of DAL (defined as final deposition of child feces into any latrine), and of DIL (defined as final deposition of child feces in a “limited,” “basic” or “safely managed” sanitation facility based on JMP ladder).

	Survey-Reported Disposal Site							Latrine Disposal	
	Used latrine	Put/rinsed into latrine	Put/rinsed into drain or ditch	Thrown in garbage	Buried	Left in open/not disposed of	Other	DAL	DIL
Afghanistan	21.5	13.3	16.6	15.4	9.1	22.1	2.0	34.8	30.6
Algeria	13.9	4.3	1.8	78.9	0.2	0.2	0.8	18.1	17.4
Angola	2.5	25.0	0.0	56.1	3.9	8.2	4.3	27.5	22.7
Bangladesh	9.1	40.2	29.5	13.3	0.6	7.1	0.3	49.3	44.1
Benin	0.6	30.8	1.9	58.5	3.4	3.2	1.6	31.4	18.3
Burundi	0.9	72.8	6.4	6.5	4.7	3.6	5.1	73.7	37.3
Cameroon	1.4	63.6	9.1	21.1	1.1	3.6	0.1	65.1	36.9
Central African Republic	3.7	43.3	10.1	28.0	2.7	9.8	2.5	47	12.3
Chad	0.8	12.6	6.1	47.5	10.3	20.7	2.1	13.4	7.2
Costa Rica	16.2	4.6	1.7	75.2	1.6	0.1	0.6	20.8	20.3
Cuba	31.3	56.9	4.9	6.3	0.1	0.5	0.1	88.2	77.8
Democratic Republic of the Congo	1.9	57.5	14.1	15.5	3.9	4.1	3.0	59.4	20.7
Ethiopia	0.7	36.2	3.7	18.3	2.8	25.5	12.8	36.9	5.2
Ghana	2.5	20.7	7.4	54.6	7.1	3.9	3.9	23.1	17.0
Guinea	1.9	52.9	8.8	26.5	3.1	6.8	0.0	54.8	30.8
Haiti	0.4	63.5	4.6	21.4	3.0	5.5	1.6	63.9	37.2
India	22.0	12.7	5.3	14.2	1.5	43.7	0.5	34.8	28.4
Indonesia	7.6	38.0	14.6	32.8	3.3	0.6	3.1	45.6	38.5
Iraq	8.6	7.3	1.7	80.0	0.3	1.6	0.6	15.8	14.3
Lesotho	5.0	47.1	4.1	16.0	7.9	17.2	2.7	52.1	45.6
Madagascar	2.0	24.1	2.9	7.6	4.5	53.5	5.3	26.1	7.0
Malawi	3.3	80.2	7.9	4.1	2.0	2.1	0.5	83.5	69.1
Maldives	5.1	4.0	0.5	89.3	0.4	0.0	0.6	9.1	8.2
Mongolia	3.4	47.0	3.8	34.2	2.1	6.3	3.3	50.4	47.7
Myanmar	23.5	36.2	18.1	7.0	2.8	12.1	0.4	59.7	34.2
Nepal	19.9	50.5	3.1	15.0	0.1	10.5	1.0	70.3	68.8
Nigeria	1.8	53.1	8.0	30.5	1.6	4.5	0.5	54.9	31.8
Pakistan	4.0	31.7	15.0	44.3	0.3	4.4	0.3	35.7	32.0
Philippines	4.0	6.2	5.5	74.4	7.4	1.2	1.3	10.2	9.1
Sao Tome and Principe	8.4	8.4	11.0	33.3	7.1	28.0	3.8	16.8	14.5
Senegal	0.3	60.9	1.7	33.5	1.4	1.0	1.1	61.2	47.0
Sierra Leone	1.8	60.2	16.5	17.4	2.2	1.9	0.0	62.1	35.7
South Africa	4.1	11.6	4.3	77.0	2.2	0.4	0.4	15.7	10.0
State of Palestine	21.0	4.1	0.5	74.3	0.0	0.1	0.0	25.1	24.5
Suriname	6.8	5.3	2.7	80.1	2.4	1.6	1.0	12.1	11.6
Tanzania	1.1	66.8	6.1	9.1	4.0	6.3	6.6	67.9	21.8
The Gambia	5.1	72.7	4.5	16.2	0.8	0.6	0.1	77.8	44.3
Timor-Leste	9.6	15.8	5.2	20.0	2.5	46.9	0.1	25.5	19.1
Tunisia	11.1	4.0	2.1	81.0	0.3	0.8	0.8	15	14.8
Uganda	2.5	73.8	9.4	5.5	4.5	4.2	0.0	76.3	27.2
Zambia	1.4	72.3	8.8	8.9	4.9	0.9	2.8	73.7	39.3
Zimbabwe	3.9	60.2	3.7	9.2	16.0	5.6	1.3	64.2	54.3
Overall	**13.2**	**27.0**	**8.4**	**23.6**	**2.3**	**23.9**	**1.5**	**40.3**	**29.0**

### 3.3 Child latrine use by age

**Fig. 3:**
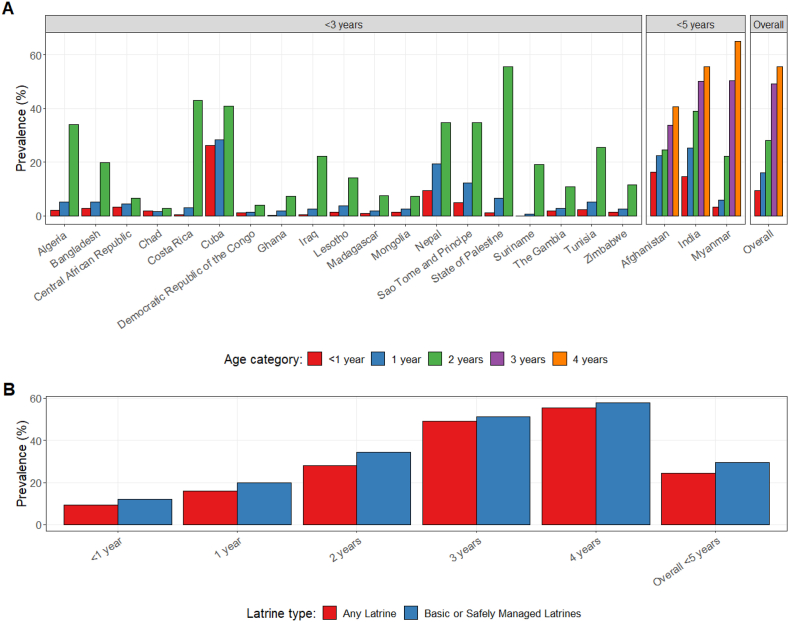
Prevalence of direct latrine use among the child's age categories (in years) (A) for any latrine shown by country, and (B) comparing child latrine use in any latrine or a basic/safely managed latrine given access to either. Data for 3-year-old and 4-year-old children are only available from India, Myanmar, and Afghanistan. All prevalence values calculated as frequency among households with access to any or a basic or safely managed latrine, respectively. Only countries which asked the CFD question of children up through at least 2 years old were included (all MICS datasets and India, Myanmar, and Afghanistan from DHS). Percentages are shown using denormalized weights. All values are shown in Table S4. (For interpretation of the references to colour in this figure legend, the reader is referred to the Web version of this article.)

Child latrine use for defecation was generally low, but increased with child age. Among all children with access to a latrine, 24.5% were reported to use it directly, compared to 29.4% of children with access to a basic or safely managed latrine who were reported to use it directly. Direct latrine use increased from 9.4% among those <1-year-old to 55.5% among 4-year-olds for any latrine type, and from 12.1% among those <1-year-old to 57.9% among 4-year-olds for improved latrines (Fig. 3A). The positive trend of latrine use and age was consistent across countries. However, there was considerable variation among countries in both absolute values and strength of the trend. Among those with access to any latrine, prevalence ranged from a low of 2.8% of 2-year-old children directly using a latrine in Chad, to a high of 55.5% in the State of Palestine, followed by India which reported 38.9%. Among children with access to a basic or safely managed latrine, the proportion of direct use was slightly higher at all ages than use of any latrine, and showed the same increasing trend as children got older (Fig. 3B). There was also considerable variation in both absolute values and the strength of the trend (Fig. S2). There was little variation in access to facilities across age groups (Table S5).

### 3.4 Determinants of child feces disposal practices among all households

In the adjusted multiple Poisson regression model, the factors associated with disposal in any latrine (Analysis 1) were generally similar to those associated with disposal in an improved latrine (Analysis 2), with a few exceptions. Across both models, wealth was the strongest predictor of feces disposal practices, although the effect of wealth was higher in the improved disposal model. As compared the poorest quintile, each successive quintile from poorer to richest were each associated with a DAL increase of 8%, 19%, 31%, and 41%, respectively (aPR = 1.08, 1.05–1.11; aPR = 1.19, 1.16–1.23; aPR = 1.31, 1.27–1.35; aPR = 1.41, 1.36–1.46, Table 3). Similarly for improved disposal, each successive quintile were each associated with successively greater DIL increases compared to the poorest quintile, with the highest increase seen for the richest quintile (aPR = 5.48, 5.19–5.78, Table 3).

**Table 3:** Adjusted prevalence ratios (aPR), 95% confidence interval (CI), and *p* values from multiple Poisson regression of predictor variables (reference categories in parentheses) on disposal in any latrine (DAL) and disposal in an improved latrine (DIL), accounting survey design and using de-normalized weighting, countries included as fixed effects (shown in Tables S6 and S7).

	Levels	Disposal in any latrine			Disposal in improved latrine		
*Variable(referent)*		aPR	95% CI	p	aPR	95% CI	p
***Child Sex****(Female)*	*Male*	0.99	(0.98–1.001)	0.075	0.997	(0.98–1.014)	0.748
***Child Age****(In* y*ears)*		**1.19**	**(1.18**–**1.2)**	**< 0.001**	**1.20**	**(1.19**–**1.22)**	**< 0.001**
***Mother Age****(In years)*		**1.001**	**(1**–**1.002)**	**0.046**	**1.004**	**(1.003**–**1.006)**	**< 0.001**
***Urbanicity****(Rural)*	*Urban*	**1.11**	**(1.083**–**1.13)**	**< 0.001**	**1.23**	**(1.2**–**1.27)**	**< 0.001**
***Breastfeeding****(Not breastfeeding)*	*Yes*	**0.95**	**(0.93**–**0.96)**	**< 0.001**	**0.94**	**(0.92**–**0.96)**	**< 0.001**
***Mother's Education****(Less than primary)*	*Primary*	1.02	(0.99–1.05)	0.13	**1.17**	**(1.12**–**1.209)**	**< 0.001**
	*Secondary or higher*	0.99	(0.97–1.02)	0.528	**1.18**	**(1.14**–**1.23)**	**< 0.001**
***Number of children under 5****(1)*	*2 or more*	**1.03**	**(1.01**–**1.05)**	**< 0.001**	**1.03**	**(1.01**–**1.05)**	**0.003**
***Number of persons in the household****(<5)*	*6 or more*	0.99	(0.97–1)	0.054	**0.96**	**(0.94**–**0.98)**	**< 0.001**
***JMP Water Ladder****(Surface and unimproved)*	*Limited*	**0.95**	**(0.91**–**0.99)**	**0.008**	**0.86**	**(0.8**–**0.92)**	**< 0.001**
	*Basic*	0.97	(0.94–1.01)	0.14	**1.09**	**(1.02**–**1.15)**	**0.006**
	*Safely Managed*	0.99	(0.98–1.05)	0.384	**1.38**	**(1.3**–**1.46)**	**< 0.001**
***JMP Sanitation Ladder****(Open defecation)*	*Unimproved*	**1.18**	**(1.09**–**1.28)**	**< 0.001**	–	–	–
	*Limited*	**1.15**	**(1.06**–**1.25)**	**0.001**	–	–	–
	*Basic/Safely Managed*	**1.18**	**(1.1**–**1.27)**	**< 0.001**	–	–	–
***Wealth quintiles****(Poorest 20%)*	*Poorer*	**1.08**	**(1.05**–**1.11)**	**< 0.001**	**2.12**	**(2.01**–**2.24)**	**< 0.001**
	*Middle*	**1.19**	**(1.16**–**1.23)**	**< 0.001**	**3.29**	**(3.12**–**3.46)**	**< 0.001**
	*Richer*	**1.31**	**(1.27**–**1.35)**	**< 0.001**	**4.59**	**(4.36**–**4.84)**	**< 0.001**
	*Richest*	**1.41**	**(1.36**–**1.46)**	**< 0.001**	**5.48**	**(5.19**–**5.78)**	**< 0.001**
***Shared Latrine****(Not shared)*	*Shared*	**0.95**	**(0.91**–**0.98)**	**0.006**	–	–	–

Reference categories: Female, Rural, Non-breastfeeding, Less than primary education, 0–1 children under 5, 1–5 people in the household, Surface and unimproved, Open defecation, Poorest 20%, and Non-shared sanitation facility.

Other factors with stronger associations and effect sizes in at least one the two models were child age, urbanicity, current breastfeeding of the child, mother's education level, level of water access, and level of sanitation access. Older children were more likely to exhibit both DAL and DIL, with an increase in DAL of 19% and an increase in DIL of 20% associated with each year increase in age (aPR = 1.19, 95% CI: 1.18–1.2; aPR = 1.2, 95% CI: 1.19–1.22, respectively; Table 3). Living in an urban environment was associated with an 11% DAL increase over rural environments (aPR = 1.11, 95% CI: 1.08–1.13; Table 3), and a greater DIL increase of 23% over rural environments (aPR = 1.23, 95% CI: 1.2–1.27; Table 3). Being a child currently breastfeeding, regardless of other food consumed, was associated with a DAL decrease of 5% over children not breastfeeding (aPR = 0.95, 95% CI: 0.93–0.96; Table 3), and was associated with a similar DIL decrease of 6% over children not breastfeeding (aPR = 0.94, 95% CI: 0.92–0.96; Table 3). Mother's education level was not associated with DAL, but it was associated with DIL, with a DIL increase of 17% associated with primary education and an 18% increase associated with secondary or higher education as compared to less than primary education (aPR = 1.17, 95% CI: 1.12–1.21; aPR = 1.18, 95% CI: 1.14–1.23, respectively; Table 3). The household's water source was more strongly associated with DIL than DAL, such that compared to surface water and unimproved sources, limited water sources were associated with a 14% DIL reduction (aPR = 0.86, 95% CI: 0.8–0.92), basic water sources associated with a 9% DIL increase (aPR = 1.09, 95% CI: 1.02–1.15), and safely managed water sources associated with a DIL increase of 38% (aPR = 1.38, 95% CI: 1.3–1.46; Table 3). Compared to open defecation, having household access to a sanitation facility classified as ‘unimproved’ was associated with a DAL increase of 18% (aPR = 1.18, 1.09–1.28; Table 3), ‘limited’ facility access was associated with a DAL increase of 15% (aPR = 1.15, 1.06–1.25; Table 3), and ‘basic or safely managed’ facility access was associated with a DAL increase of 18% (aPR = 1.39, 1.1–1.27; Table 3). However, sharing household sanitation facilities was associated with a DAL decrease of 5% as opposed to not sharing facilities (aPR = 0.95, 0.91–0.98; Table 3).

Other factors with weak associations or effect sizes included mother's age, number of children under five years in the household, and number of persons in the household. Child sex was not significantly associated with DIL or DAL (Table 3).

### 3.5 Determinants of child feces disposal practices among households with access to sanitation

Restricting analyses to only observations for children with household access to a latrine of any kind (new n = 2270, 885 observations, DAL as the response, Analysis 3) or an improved latrine (new n = 222,097 observations, DIL as the response, Analysis 4) indicated that only 52.2% of observations with household access to any latrine engaged in DAL, and among those with access to an improved facility, only 51.5% engaged in DIL. Household access to latrines of any kind and improved latrines were highly variable between countries, as were their respective uses for CFD conditional on household access (Fig. S3). The models indicated overall quantitatively similar results to the main analysis, especially for DAL (see Table S8). The magnitude of some associations with DIL differed, including a slight decrease in the association with urbanicity and DIL (from aPR = 1.23, 1.2–1.27, to aPR = 1.10, 1.07–1.12; Table S8), a decrease to non-significant association of mother's education and DIL (from 18% increases associated with primary and secondary or higher education compared to less than primary), and weaker but still significant positive associations between DIL and wealth quintiles (from over 200% increases to 13%, 26%, 39%, and 50% increases associated with each successive quintile compared to the poorest; Table S8). The direction of a single association changed: a switch from a 9% increase in DIL to an 8% decrease versus surface and unimproved water (Table S8) and a non-significant association between DIL with safely managed water (from a 38% increase versus surface and unimproved water; Table S8).

### 3.6 Sensitivity analyses

Results from sensitivity analyses indicate the main results were highly robust across the different permutations of the analysis (see Tables S9 and S10 for DAL and DIL, respectively). The main analysis and the sensitivity analysis model results using three different methods to control for the differences between DHS and MICS (i.e. dropping children over 2 years old, using only the youngest child under 2, and including a mother clustering term) were nearly identical to one another. The results of models that weighted countries equally or included all countries except India were similar to one another, and overall the results were similar to those of the main analysis, although the association between wealth quintiles and both DAL and DIL were weaker.

## 4 Discussion

This study used a large, nationally representative dataset from 42 LMICs to explore the scope of child feces disposal practices and determinants of proper disposal in a latrine. Results showed that proper disposal in a latrine was low overall, and not explained by latrine access alone but instead influenced by several factors. Child latrine use was also low and highly variable for children of the same age across countries, suggesting that children in many LMICs are likely developmentally ready to use the latrine at younger ages than initiated. These results can inform the scope and enabling factors to safe child feces disposal practices in LMICs.

### 4.1 Prevalence of child feces disposal practices

Across the LMICs surveyed, the prevalence of child feces disposal in a latrine was low, at only forty percent, and lower for disposal in improved latrines at only twenty-nine percent. Despite gains in WASH coverage over the past few decades (UNICEF/WHO, 2021), there are still many children (and their families) at risk of being exposed to pathogens from unsafe child feces disposal practices. There was considerable heterogeneity among countries in the prevalence of disposal of child feces in latrines. Among the LMICs surveyed, countries in central and eastern Africa showed the highest prevalence of DAL, though many of these same countries showed lower relative prevalence of DIL, likely due to lower levels of access to improved latrines (Table S3). North African countries and Afghanistan showed low DAL and DIL overall. Caribbean and Latin American coverage was limited, but showed a mix of both DAL and DIL prevalence in the region. Even within these regions, considerable variation was observed here and elsewhere (Seidu et al., 2021). While much of this variation may be due to differences in sanitation access and behavioral and normative factors around child feces disposal across countries and regions, solid waste infrastructure may also play a role in this variation. Wealthier countries with more developed solid waste infrastructure may enable more contained disposal of child feces with solid waste, making that disposal alternative more desirable to households than disposal in a latrine. However, sufficient information on solid waste infrastructure was not included in these datasets, so could not be assessed as part of this analysis.

The prevalence values of safe CFD from individual countries are similar to those reported elsewhere (Azage and Haile, 2015; Nkoka, 2020; Sahiledengle, 2020; Seidu et al., 2021). While few reports have aggregated many countries in this manner, the considerable between country variation in DAL and DIL prevalence observed here has been noted elsewhere as well (Seidu et al., 2021). This variation may reflect factors operating at national or community levels such as historical and cultural attitudes (Novotný et al., 2017), political and economic stability (Als et al., 2020), and differences in public health messaging and WASH infrastructure investment (Beardsley et al., 2021). While further work should explore inter-related effects of factors across national, community, and household scales, the factors in the present analysis focus only on household and individual level attributes common across countries.

Child feces management improvements may have an even greater impact in countries with a relatively larger proportion of their population under 5 years old, such as Afghanistan, Angola, Madagascar, and Ethiopia, where greater than 15% of the total population is under 5 years old (UN World Population Prospects, 2020). These countries each exhibited a prevalence of child feces disposal in any latrine less than 40%, and efforts in these large countries to increase adequate CFD behavior should be highlighted.

### 4.2 Factors associated with child feces disposal practices

Disposal of child feces in latrines increased with age, consistent with prior work (Nkoka, 2020; Sahiledengle, 2020; Seidu et al., 2021). Increased direct latrine use amongst older children may contribute to this, however a slightly larger effect was observed after restricting the ages to less than 2-years-old, meaning that even among 0–1 and 1-2-year-olds the effect holds. Some caregivers may believe their very young children's feces is not harmful (Majorin et al., 2014), making them less likely to dispose of this feces in latrines. This may also explain the observed negative association between current breastfeeding and disposal of child feces in latrines. The potential for older, ambulatory children to spread fecal pathogens through unsafe CFD can be greater than in young children, so while this trend is positive, there is still considerable room for improvement, especially among older children under 5 years old. Early interventions may be critical in this effort to avoid potential habit forming among young children openly defecating (Bauza et al., 2020). Child sex was not associated with either DAL or DIL, consistent with other multinational studies (Seidu et al., 2021), however sex-specific considerations in public health interventions may still be warranted.

The positive associations between mother's age and DAL and DIL were extremely small, and coupled with other null findings (Azage and Haile, 2015), suggests this variable holds little relevance to public health. Interestingly, the mother's education was not associated with DAL, but there was an increase of DIL associated with mother's education level being anything greater than primary education. Improvements to safe CFD with mother's education is consistent with work in individual countries (Nkoka, 2020; Sahiledengle, 2020), and multiple country assessments (Beardsley et al., 2021). Educated mothers may be more likely to understand the importance of safe CFD practices for disease control (Dreibelbis et al., 2013; Mwambete and Joseph, 2010), and child caretakers (often women) are typically responsible for CFD, suggesting that expanding women's education could increase appropriate CFD as well as other beneficial health and well-being outcomes.

Urban living was associated with modest DAL and DIL increases over rural living, which has been observed in Ethiopia (Azage and Haile, 2015; Sahiledengle, 2020). Access to latrines, and improved latrines, tends to be greater in urban areas (UNICEF/WHO, 2021), and open defecation may be socially unacceptable in more urban areas.

The strongest factor associated with both DAL and DIL was the intra-country relative household wealth, which is frequently observed in studies of CFD (Azage and Haile, 2015; Sahiledengle, 2020), and may be related to either greater access to latrines themselves, or information regarding the safety, importance, and methods for safe CFD (Nkoka, 2020), or residual confounding (Majorin et al., 2019a).

Neither ‘basic’ nor ‘safely managed’ water sources were associated with a difference in DAL compared to ‘surface water’ or ‘unimproved’ sources, however they were associated with minor and modest DIL increases, respectively. This likely reflects that household access to these higher categories of improved water sources often accompanies access to improved sanitation. The minor reduction in DAL, and the stronger reduction in DIL associated with ‘limited’ water sources may reflect that ‘limited’ water sources are improved but more than 30 min round-trip from the home. Not having nearby access to water may accompany a lack of nearby access to sanitation facilities as well, resulting in a barrier to using those latrines for CFD, even if used by adults, as reported in one study in Odisha, India (Majorin et al., 2019a). Water access is also important for caregiver handwashing after CFD, cleaning any tools used to handle child feces, and flushing the latrine, if applicable. In this dataset, a quarter of households with safely managed water reported open defecation or unimproved latrines, implying there is still considerable disparity in sanitation after accounting for water source improvements. These results suggest that improvements to CFD will not *necessarily* come from improving water sources without also improving sanitation, but slight improvements may come from making access to water easier, which may accompany increasing sanitation facility access in cost effective ways. These findings are consistent with prior null associations from Ethiopia (Azage and Haile, 2015), and one study which found non-significant associations in Ethiopia, India, and Zambia (Beardsley et al., 2021).

### 4.3 Access to sanitation facilities

While over half of children in these LMICs were from households with access to improved latrines, access to sanitation facilities does not necessarily correspond to their use for CFD. Indeed, just half of those with access to a latrine actually dispose of their child's feces in it, and the same pattern holds for improved facilities, meaning there are other barriers or knowledge gaps to using latrines for disposing of child's fecal waste. DAL was modestly associated with access to a sanitation facility of any kind, however the specific type of facility (unimproved, limited, or basic/safely managed) does not appear to make a difference. Sanitation facilities higher on the JMP ladder do not appear to increase its use for CFD, as evidenced by the fact that the aPR's for each of the JMP sanitation ladder categories were almost exactly the same in our model, consistent with one study from Bangladesh (George et al., 2016), but in contrast to other studies which found that improved latrines were associated with increased safe CFD (Azage and Haile, 2015; Beardsley et al., 2021; Majorin et al., 2019a; Sahiledengle, 2020). These findings suggest that to increase safe CFD, expanding availability of any kind of latrine may be sufficient, as long as the latrine is able to contain the fecal waste.

When analyses were restricted to observations for children with household access to a latrine, the model of DAL was almost exactly the same, and DIL showed minor magnitude changes. These findings indicate that our analyses identify determinants of child feces disposal practices instead of latrine access, and underscore the notion that for CFD in any kind of latrine, the associated factors described here operate independently of access, and access alone is insufficient to drive CFD behavior. The two associations with DIL that changed in this sub-analysis were associations between DIL and a basic and safely managed water sources compared to surface and unimproved water, and the strength of the association of DIL with wealth quintiles. These patterns highlight that once improved latrine access is accounted for, water sources have little effect on CFD, and that even among those with improved latrine access, relative wealth is an important factor associated with CFD.

There was a slight reduction in DAL associated with facilities being shared, consistent with findings from Ghana and India (Majorin et al., 2019a; Ritter et al., 2018). This reduction may also be due to children being less likely to use shared facilities, which may sometimes be less clean than private latrines or have a fee associated with use. With respect to increasing CFD coverage, further cost-benefit analyses may be warranted to evaluate interventions seeking to improve sanitation facility coverage and the ratio of households to sanitation facilities built.

Flush toilet infrastructure and public sewage can be prohibitively expensive in rural and low-income regions, however in such settings basic and safely managed sanitation under JMP standards can be achieved using improved latrines. Even among the fifteen countries included here with greater than 50% of households having access to flush or pour-flush toilets, the mean use of such facilities for CFD was less than 35%, further indicating that facility access alone does not determine usage for CFD.

### 4.4 Child latrine use

Levels of child latrine use were low and highly variable across countries for children of the same age. Despite considerable variation in prevalence across countries, there was a positive trend of increasing direct latrine use with age within a country that was consistently observed. However, less than two-thirds of surveyed children with access to latrines are using them directly by 5 years old including only one-third of 2-year-olds, and more concerted efforts to improve this behavior are warranted to limit fecal contamination (Bauza et al., 2020), especially in sub-Saharan Africa. Latrines that are improved appear to increase this behavior slightly, however the direction of causality is unclear and latrine use among children with access to improved latrines was still low. The high variability of 2-year-old children using the latrine from 2.8% in Chad to a 55% in the State of Palestine, suggests that children are likely developmentally ready to use a latrine by this age, but there are likely other barriers to this behavior. Further research into the factors that influence this behavior are warranted, as well as interventions designed to reduce these barriers and enable younger child latrine training and use (Bauza et al., 2020; Sclar et al., 2022).

Currently, DHS only collects data on CFD practices for children under two, assuming that children's sanitation practices can be accounted for by the overall household sanitation practice (such as latrine use) once they are two years old. However, our analysis demonstrates that this is unlikely, and CFD practices for children that are two years and older are needed to better understand sanitation practices of a household and potential interventions to limit exposure to sanitation-related pathogens. This is especially critical amid calls for ‘transformative WASH’ interventions that ‘radically reduce fecal contamination in the household environment’ in order to realize health benefits (Pickering et al., 2019). While DHS used to collect CFD information for the youngest child under five years in a household, this was changed to the youngest children under two years with the release of the DHS-VII version of the survey without explanation. Our results present a strong case that DHS and MICS surveys should include all children under 5 years old when asking about CFD. While this may slightly increase survey time, there is great insight to be gained from richer data on children up to 5 years old, which can be used to better tailor behavioral and health interventions beyond just child feces management.

### 4.5 Sensitivity analyses

The similarity of results from the main analysis to the three models controlling for differences between DHS and MICS suggest that these discrepancies are minor, and more importantly that the trends in this analysis are robust. While the two weighting methods (denormalized and equal-country) provided qualitatively similar results, the denormalized weighting method used in the main analysis is more representative of the total population surveyed and therefore more representative of the target population of all children in LMICs. The analysis was robust to the over-representation of India in the dataset, as the model without India was very similar to the main results. One factor that decreased in magnitude was the association between intra-country relative wealth quintile and DAL and DIL, suggesting that while there were still strong associations with these wealth quintiles, the magnitude in the main dataset may be influenced by India, where wealth inequality is exceptionally high.

### 4.6 Limitations

Because this is a large pooled analysis, the exact values of the associations reported here may not exactly reflect those observed in a single country, but they benefit from greater generalizability to LMICs globally. The analysis is representative of millions of children from 42 different countries and multiple world regions, providing strong insight on common global trends. The cross-sectional nature of the study limits causal inference, but the broad scope again highlights global trends. Additionally, as child feces disposal practices were self-reported, this could have introduced reporting bias and potential overreporting of perceived desirable hygienic behaviors (Curtis et al., 1993; ManuN'ebo et al., 1997). If this reporting bias exists in our dataset, our estimates would be likely overestimate safe CFD, meaning the true levels could be even lower than our estimates.

Deposition of child feces via disposable diapers or some other methods into the garbage could be adequate for handling child feces, depending on solid waste infrastructure and storage and handling methods. However, such analysis is limited because only MICS records information on solid waste infrastructure, and is further limited by respondent knowledge. Therefore, the decision to classify solid waste disposal as failing to meet JMP ‘improved’ or ‘appropriate’ guidelines may have introduced differential mis-classification bias with respect to wealthier countries or areas with good solid waste management practices. The expense of disposable diapers suggests only wealthier families are likely to use them, and such infrastructure is often limited to wealthier countries or neighborhoods, and therefore potential mis-classification would bias the effect of wealth toward the null. Solid waste disposal accounted for 23.6% of this dataset, and inadequate management of solid waste is common in LMICs, so the total bias was not likely very strong.

While children with disabilities may have difficulty using a latrine, information on child disability is extremely limited in DHS and MICS datasets, limiting our ability to discuss a possible role that disability may have in child feces disposal and latrine use practices.

## 5 Conclusions

The present analysis of a large nationally representative dataset for 42 LMICs demonstrates that adequate child feces disposal in a latrine and child latrine use were generally low and highly geographically variable. Only half of children with household access to any latrine or improved latrines had their feces disposed of in such facilities, indicating factors other than access drive child feces disposal behavior. Disposal of child feces in a latrine increased with child age, urban settings, higher income, and improved water or sanitation sources, and mother's education only was associated with increased disposal in improved latrines. These results were also highly robust across several sensitivity analyses. Additionally, few children had transitioned to latrine use by age two, indicating the importance of collecting CFD data for older children, particularly in nationally representative surveys like DHS. Overall, children's feces in LMICs are infrequently disposed of in any latrine type, and even less frequently in improved latrines. In order to minimize health risks in LMICs, increased effort must be undertaken not just to increase sanitation coverage but to address these common barriers to safe child feces disposal and child latrine use.

## Funding

This work was funded in part by support for SGM from the Laney Graduate School, 10.13039/100006939Emory University, and in part by the 10.13039/100000865Bill & Melinda Gates Foundation [NV 008372].

## Data availability

Data are freely available and accessible through written permission of DHS and MICS programs.
