# Supplementary material for: Global practices, geographic variation, and determinants of child feces disposal in 42 low- and middle-income countries: An analysis of standardized cross-sectional national surveys from 2016 – 2020
Source: Int J Hyg Environ Health. 2022 Aug;245:114024. doi: 10.1016/j.ijheh.2022.114024 (PMC9489922; doi:10.1016/j.ijheh.2022.114024)

**Supplemental Tables and Figures for “Global practices, geographic variation, and determinants of child feces disposal in 42 low- and middle-income countries: an analysis of standardized cross-sectional national surveys from 2016-2020”**

**Results**

**Table S1.** Overall distributions (shown as percentages) of personal and household data using denormalized weights, and mean (SD) for continuous variables

| Variable | Level | Weighted Prevalence (%) |
| --- | --- | --- |
| Child Sex | Female ^1,4^ | **47.6** |
| Urbanicity | Rural ^1,2,4^ | **66.9** |
| Breastfeeding | Breastfeeding ^3,4^ | **65.8** |
| Mother Education | Less than primary ^1^ | **28.5** |
|  | Primary | **20.5** |
|  | Secondary or Higher | **51.0** |
| Persons in household | 1-5 ^1^ | **44.3** |
|  | 6 or more | **55.7** |
| Children under 5 in household | 0-1 ^1^ | **48.3** |
|  | 2 or more | **51.7** |
| Wealth Index Quintile | Poorer ^4^ | **21.1** |
|  | Middle | **20** |
|  | Richer | **19.1** |
|  | Richest | **17.1** |
| Child Age Mean (SD) |  | **1.16 (1.2)** |
| Mother Age Mean (SD) |  | **27.7 (6.0)** |
| JMP Sanitation Ladder | Open Defecation ^1^ | **29.3** |
|  | Unimproved | **12.9** |
|  | Limited | **13** |
|  | Basic/Safely Managed | **44.8** |
| Shared sanitation facility | Shared ^4,5^ | **24.1** |
| JMP Water Ladder | Surface and Unimproved ^1^ | **13.6** |
|  | Limited | **17.8** |
|  | Basic | **24.7** |
|  | Safely Managed | **43.9** |

*^1^ A reference category for regression analysis*

*^2^ Rural includes both rural and ‘camp’ definitions from DHS data (‘camp’ seen only in Suriname and Palestine, n = 908 respondents total)*

*^3^ Breastfeeding designates any breastfeeding, regardless of other food fed to the child*

*^4^ Unlisted categories include ‘male’ (Child sex), ‘Urban’ (Urbanicity), ‘Not breastfeeding’ (Breastfeeding), ‘Poorest’ (Wealth Quintile), and ‘Not shared’ (Shared sanitation facility)*

*^5^ 29% of the denormalized data are missing a response to this question, however this is largely comprised of open defecators, for whom this question was omitted during survey administration*

**Table S2.** Distributions of personal and household descriptive data by country, percentages shown using denormalized weights, and mean (SD) calculations for continuous variables

| **Country** | **Child Sex** | **Urbanicity** | **Breast feeding** | **Mother Education** | | | **Persons in household** | | **Children under 5 in household** | | **Wealth Index Quintile** | | | | **Mean Child Age** | **Mean Mother Age** |
| --- | --- | --- | --- | --- | --- | --- | --- | --- | --- | --- | --- | --- | --- | --- | --- | --- |
|  | **Female^1^** | **Rural^1^** | **Breast feeding^1^** | **Less than primary** | **Primary** | **Secondary or Higher** | **1-5^1^** | **6 or more** | **0-1^1^** | **2 or more** | **Poorer^1^** | **Middle** | **Richer** | **Richest** | **Mean (SD)** | **Mean (SD)** |
| **Afghanistan** | 48.1 | 76.6 | 16.8 | 82.8 | 8.2 | 9.0 | 16.5 | 83.5 | 28.7 | 71.3 | 20.2 | 20.2 | 20.8 | 18.9 | 1.4 (1.2) | 28.9 (6.9) |
| **Algeria** | 48.7 | 43.4 | 45.4 | 11.2 | 14.2 | 74.6 | 54.6 | 45.4 | 51.3 | 48.7 | 23.5 | 19.8 | 18.5 | 14.8 | 1.01 (0.8) | 31.9 (5.9) |
| **Angola** | 50.3 | 39.6 | 80 | 28.3 | 38.7 | 33.0 | 44 | 56 | 27.0 | 73.0 | 23.6 | 21.8 | 17.6 | 15 | 0.46 (0.5) | 26.8 (7.0) |
| **Bangladesh** | 47.8 | 78.5 | 82.1 | 9.5 | 23.3 | 67.2 | 61.6 | 38.4 | 73.7 | 26.3 | 19.3 | 18.6 | 19.8 | 21 | 1.00 (0.8) | 26.4 (5.9) |
| **Benin** | 49.7 | 61.6 | 83.6 | 62.8 | 18.7 | 18.5 | 39.3 | 60.7 | 25.3 | 74.7 | 20.5 | 20.8 | 19.9 | 17.9 | 0.45 (0.5) | 27.8 (6.5) |
| **Burundi** | 49.5 | 91.0 | 93.6 | 43.5 | 44.4 | 12.1 | 48.3 | 51.7 | 28.7 | 71.3 | 22 | 20.8 | 19 | 16.5 | 0.49 (0.5) | 29.4 (6.5) |
| **Cameroon** | 47.4 | 56.6 | 68 | 27.1 | 30.6 | 42.3 | 32.6 | 67.4 | 26.8 | 73.2 | 23.3 | 20.8 | 17.9 | 15.1 | 0.47 (0.5) | 27.1 (6.5) |
| **Central African Republic** | 50.9 | 70.6 | 68.2 | 40.8 | 42.7 | 16.5 | 33.3 | 66.7 | 27.6 | 72.5 | 22 | 21.8 | 19 | 14.8 | 0.96 (0.8) | 27.9 (7.1) |
| **Chad** | 49.4 | 84.5 | 67.1 | 64.7 | 22.0 | 13.4 | 32.4 | 67.6 | 25.9 | 74.1 | 21.5 | 21 | 20.3 | 16.3 | 0.99 (0.8) | 28.2 (6.9) |
| **Costa Rica** | 48.1 | 28.9 | 48.1 | 0.8 | 19.3 | 80.0 | 73.5 | 26.5 | 70.5 | 29.5 | 21.7 | 17.7 | 17.6 | 14.9 | 1.06 (0.8) | 28.2 (6.1) |
| **Cuba** | 48.3 | 35.9 | 36 | 0.0 | 0.9 | 99.1 | 79.3 | 20.7 | 78.9 | 21.1 | 19.4 | 17.3 | 20 | 21.7 | 0.95 (0.8) | 27.9 (6.1) |
| **Democratic Republic of the Congo** | 50.8 | 61.2 | 64.4 | 17.9 | 33.6 | 48.5 | 40.5 | 59.5 | 30.0 | 70.0 | 22.1 | 19.4 | 19 | 15.7 | 0.97 (0.8) | 29.1 (7.0) |
| **Ethiopia** | 52.5 | 88.0 | 90 | 60.3 | 30.9 | 8.8 | 46.3 | 53.7 | 37.8 | 62.2 | 21.8 | 20.9 | 18.2 | 15.5 | 0.46 (0.5) | 28.4 (6.6) |
| **Ghana** | 50.1 | 56.6 | 59.6 | 23.3 | 20.9 | 55.8 | 41.9 | 58.1 | 43.9 | 56.1 | 20.5 | 19.9 | 19.7 | 19 | 0.98 (0.8) | 30.1 (7.1) |
| **Guinea** | 48.9 | 71.3 | 85.1 | 74.3 | 11.9 | 13.8 | 28.9 | 71.1 | 24.7 | 75.4 | 22.5 | 19.6 | 18.5 | 15.7 | 0.46 (0.5) | 27.8 (7.1) |
| **Haiti** | 50.5 | 67.4 | 74.8 | 17.4 | 39.4 | 43.2 | 44.6 | 55.4 | 45.1 | 54.9 | 22.7 | 21.5 | 16.2 | 13.1 | 0.46 (0.5) | 28.4 (6.9) |
| **India** | 45.6 | 70.1 | 60.2 | 27.4 | 13.4 | 59.2 | 45.7 | 54.3 | 56.2 | 43.8 | 21.1 | 19.9 | 19.1 | 16.8 | 1.68 (1.4) | 26.9 (5.1) |
| **Indonesia** | 48.4 | 51.5 | 75.3 | 0.9 | 23.0 | 76.1 | 59.7 | 40.3 | 67.6 | 32.4 | 20.1 | 19.8 | 21.1 | 19.1 | 0.51 (0.5) | 29.6 (6.4) |
| **Iraq** | 48.2 | 31.5 | 40.8 | 18.6 | 43.1 | 38.3 | 25.7 | 74.3 | 35.4 | 64.6 | 21.5 | 20.7 | 18 | 17.7 | 1 (0.8) | 28.5 (6.7) |
| **Lesotho** | 50.9 | 65.5 | 43.8 | 0.0 | 35.1 | 64.9 | 58.7 | 41.3 | 69.0 | 31.1 | 20.6 | 20.4 | 19.1 | 16.4 | 1.02 (0.8) | 27.8 (7.3) |
| **Madagascar** | 48.1 | 80.1 | 72.1 | 22.3 | 48.7 | 28.9 | 55.6 | 44.4 | 51.6 | 48.4 | 22.6 | 20 | 16.9 | 15.2 | 0.95 (0.8) | 27.1 (7.4) |
| **Malawi** | 49.7 | 86.3 | 88.5 | 12.0 | 66.9 | 21.1 | 56.8 | 43.2 | 45.2 | 54.8 | 22.7 | 19.4 | 16.9 | 15.5 | 0.49 (0.5) | 26.7 (6.8) |
| **Maldives** | 50.9 | 62.8 | 82.9 | 0.9 | 15.0 | 84.1 | 23.9 | 76.1 | 46.2 | 53.8 | 21.8 | 21.9 | 17.7 | 20.3 | 0.48 (0.5) | 29.4 (5.2) |
| **Mongolia** | 49.2 | 33.3 | 65.7 | 4.2 | 3.9 | 91.9 | 66.9 | 33.1 | 56.8 | 43.2 | 19.2 | 22 | 18 | 20.5 | 1.00 (0.8) | 30.4 (6.0) |
| **Myanmar** | 48.6 | 76.5 | 53.8 | 16.2 | 45.5 | 38.3 | 55.8 | 44.2 | 71.4 | 28.6 | 21.7 | 17.5 | 17.9 | 15.6 | 1.8 (1.4) | 30.9 (6.6) |
| **Nepal** | 46.8 | 35.4 | 91.1 | 23.5 | 30.7 | 45.9 | 49.5 | 50.5 | 60.1 | 39.9 | 20.7 | 19.9 | 19.3 | 16.5 | 0.99 (0.8) | 26.1 (5.6) |
| **Nigeria** | 48.6 | 61.4 | 79.6 | 44.5 | 14.4 | 41.1 | 45.1 | 54.9 | 29.9 | 70.1 | 22.5 | 20.6 | 18.7 | 16.7 | 0.48 (0.5) | 28.3 (6.7) |
| **Pakistan** | 49.9 | 66.9 | 75.6 | 46.8 | 15.5 | 37.8 | 21.1 | 78.9 | 24.3 | 75.7 | 18.7 | 21.8 | 18.9 | 19.4 | 0.48 (0.5) | 27.8 (5.8) |
| **Philippines** | 47.2 | 55.8 | 69.4 | 1.1 | 15.5 | 83.4 | 48.8 | 51.2 | 48.0 | 52.1 | 21.8 | 19.7 | 17.4 | 14.5 | 0.49 (0.5) | 28.6 (6.6) |
| **Sao Tome and Principe** | 48.9 | 33.1 | 50.9 | 2.8 | 44.4 | 52.9 | 58.6 | 41.4 | 62.3 | 37.7 | 21.1 | 19.1 | 20.3 | 15 | 1.06 (0.8) | 28.8 (7.0) |
| **Senegal** | 52.0 | 62.6 | 84.3 | 60.1 | 19.6 | 20.4 | 8.4 | 91.6 | 15.5 | 84.5 | 23.3 | 20.2 | 16.6 | 17.4 | 0.5 (0.5) | 28.7 (6.7) |
| **Sierra Leone** | 49.2 | 65.7 | 81.2 | 52.0 | 15.7 | 32.4 | 37.9 | 62.1 | 37.2 | 62.8 | 22.4 | 20.1 | 18.5 | 15.3 | 0.48 (0.5) | 27.9 (6.9) |
| **South Africa** | 48.1 | 38.5 | 51.3 | 1.4 | 7.7 | 91.0 | 53.9 | 46.1 | 52.1 | 47.9 | 22.4 | 21.4 | 19.8 | 13.9 | 0.48 (0.5) | 27.7 (6.5) |
| **State of Palestine** | 48.3 | 23.9 | 40.1 | 0.0 | 18.0 | 82.1 | 55.4 | 44.6 | 39.0 | 61.0 | 18 | 20.5 | 21.3 | 16.5 | 1.00 (0.8) | 28.4 (5.8) |
| **Suriname** | 48.3 | 32.3 | 33.7 | 5.7 | 16.9 | 77.4 | 42.5 | 57.5 | 51.3 | 48.8 | 23.3 | 18.4 | 16.4 | 12 | 1.02 (0.8) | 28.9 (7.1) |
| **Tanzania** | 49.1 | 72.7 | 85.1 | 19.5 | 63.7 | 16.8 | 39.2 | 60.8 | 36.3 | 63.7 | 21.1 | 18.6 | 19 | 16.6 | 0.5 (0.5) | 27.5 (7.0) |
| **The Gambia** | 50.4 | 38.1 | 61.5 | 50.7 | 17.2 | 32.1 | 14 | 86 | 23.9 | 76.2 | 21.8 | 20.3 | 19.5 | 15.4 | 1.00 (0.8) | 29.1 (6.7) |
| **Timor-Leste** | 49.0 | 72.9 | 71.5 | 22.8 | 17.0 | 60.2 | 29 | 71 | 32.2 | 67.8 | 20.5 | 20.9 | 20.4 | 18.8 | 0.48 (0.5) | 28.8 (6.4) |
| **Tunisia** | 46.7 | 37.4 | 39.8 | 6.6 | 22.7 | 70.7 | 75.6 | 24.4 | 51.9 | 48.1 | 22.2 | 19.7 | 21.9 | 16.2 | 1.02 (0.8) | 32.4 (5.4) |
| **Uganda** | 49.3 | 78.8 | 83 | 9.6 | 60.7 | 29.7 | 48.3 | 51.7 | 32.7 | 67.3 | 21.3 | 19.1 | 17.5 | 19.3 | 0.46 (0.5) | 27.0 (6.6) |
| **Zambia** | 49.8 | 65.6 | 79.9 | 9.5 | 50.7 | 39.8 | 42.3 | 57.7 | 36.8 | 63.2 | 22.7 | 18.7 | 17.1 | 15.8 | 0.48 (0.5) | 27.2 (7.2) |
| **Zimbabwe** | 50.8 | 69.4 | 52.2 | 1.1 | 27.6 | 71.3 | 60.4 | 39.6 | 64.6 | 35.5 | 21.1 | 18.3 | 20.5 | 16.8 | 0.99 (0.8) | 28.6 (7.3) |
| **Overall** | **47.6** | **66.9** | **65.8** | **28.5** | **20.5** | **51.0** | **44.3** | **55.7** | **48.3** | **51.7** | **21.1** | **20** | **19.1** | **17.1** | **1.16 (1.2)** | **27.7 (6.0)** |

*^1^Reference categories in subsequent analyses: Female, Rural, Non-breastfeeding, Less than primary education, 0-1 children under 5, 1-5 people in the household, Poorest quintile*

**Table S3.** Prevalence of children with household access to each JMP ladder sanitation and water categorization (shown as percentages using denormalized weights) by country

| **Country** | **JMP Sanitation Ladder** | | | | **Shared sanitation facility** | **JMP Water Ladder** | | | |
| --- | --- | --- | --- | --- | --- | --- | --- | --- | --- |
|  | **Open Defecation ^1^** | **Unimproved** | **Limited** | **Basic/Safely Managed** | **Shared^2^** | **Surface and Unimproved^1^** | **Limited** | **Basic** | **Safely Managed** |
| **Afghanistan** | 13.9 | 8.9 | 15 | 62.2 | 19.1 | 26.4 | 8.7 | 29.9 | 35 |
| **Algeria** | 3.2 | 0.9 | 14.6 | 81.4 | 15.4 | 0.7 | 74.2 | 19.6 | 5.4 |
| **Angola** | 31 | 5.3 | 23.4 | 40.3 | 36.3 | 32 | 13.2 | 25.4 | 29.4 |
| **Bangladesh** | 4.9 | 11.3 | 24.7 | 59.1 | 30.4 | 1.5 | 82.7 | 15.3 | 0.4 |
| **Benin** | 59.4 | 13.1 | 17.4 | 10.1 | 65.7 | 32.6 | 11 | 34.8 | 21.6 |
| **Burundi** | 2.4 | 47.8 | 9.2 | 40.6 | 16.7 | 17.8 | 20.2 | 54.2 | 7.9 |
| **Cameroon** | 6.6 | 40.5 | 15.3 | 37.6 | 24.9 | 28 | 12.7 | 36.3 | 23.1 |
| **Central African Republic** | 29.8 | 51.5 | 10.2 | 8.5 | 54.2 | 44.4 | 24.3 | 30.1 | 1.3 |
| **Chad** | 71.9 | 14.1 | 4 | 10 | 29.1 | 39.9 | 16.8 | 41.8 | 1.5 |
| **Costa Rica** | 1.2 | 1 | 3.5 | 94.3 | 3.9 | 0.2 | 98.2 | 1 | 0.6 |
| **Cuba** | 2.7 | 8.7 | 3.3 | 85.4 | 4.2 | 0.8 | 90.3 | 7.7 | 1.3 |
| **Democratic Republic of the Congo** | 13.7 | 57.5 | 17.5 | 11.2 | 49.9 | 45 | 35.5 | 19.2 | 0.3 |
| **Ethiopia** | 36.4 | 53.3 | 5.2 | 5.2 | 25.1 | 41.1 | 17.5 | 30.1 | 11.3 |
| **Ghana** | 23.7 | 16.5 | 43.8 | 15.9 | 76 | 14.7 | 25.7 | 57.5 | 2.1 |
| **Guinea** | 14.8 | 37.8 | 25 | 22.4 | 51.1 | 22.5 | 22.1 | 30.2 | 25.2 |
| **Haiti** | 31.6 | 21.2 | 21.2 | 26.1 | 43.9 | 28.8 | 15.3 | 45.3 | 10.5 |
| **India** | 45 | 1.9 | 9.6 | 43.5 | 18.7 | 5.5 | 8.7 | 25.8 | 60.1 |
| **Indonesia** | 8.9 | 9.1 | 6.7 | 75.3 | 6.7 | 8.6 | 4.5 | 7.9 | 79 |
| **Iraq** | 4.8 | 1.1 | 3 | 91.1 | 3.2 | 0.5 | 76.4 | 8.9 | 14.2 |
| **Lesotho** | 23.8 | 6.9 | 25.5 | 43.8 | 35.9 | 13.2 | 41 | 45.8 | 0 |
| **Madagascar** | 44.9 | 42.3 | 8.8 | 4 | 68 | 61.4 | 12.7 | 23.7 | 2.2 |
| **Malawi** | 6.6 | 11.3 | 32.9 | 49.2 | 40.1 | 13.5 | 26.4 | 48.2 | 12 |
| **Maldives** | 0.5 | 0.4 | 0.9 | 98.1 | 0.9 | 0 | 2 | 2.1 | 95.9 |
| **Mongolia** | 8.8 | 2.9 | 20.7 | 67.6 | 23.6 | 13.7 | 36.3 | 49.2 | 0.8 |
| **Myanmar** | 13.6 | 33 | 10 | 43.4 | 20.5 | 16.9 | 4.9 | 33.5 | 44.7 |
| **Nepal** | 6.8 | 0.6 | 17.4 | 75.2 | 19 | 2.2 | 78.2 | 18.8 | 0.8 |
| **Nigeria** | 23.7 | 25 | 19.9 | 31.3 | 34.8 | 30.2 | 11.6 | 38.4 | 19.7 |
| **Pakistan** | 16.8 | 3.7 | 11.7 | 67.8 | 15.2 | 4.6 | 11.7 | 12.9 | 70.7 |
| **Philippines** | 7.4 | 2.8 | 19.5 | 70.3 | 22.1 | 4.4 | 2.7 | 16.5 | 76.4 |
| **Sao Tome and Principe** | 55.7 | 2.3 | 5.2 | 36.9 | 17.1 | 2.7 | 56.4 | 34.2 | 6.7 |
| **Senegal** | 11.8 | 13.6 | 14.4 | 60.2 | 21.1 | 15.3 | 12.2 | 8.3 | 64.2 |
| **Sierra Leone** | 21.1 | 27.7 | 36.7 | 14.5 | 73.7 | 37.9 | 9.9 | 39.3 | 12.9 |
| **South Africa** | 4.2 | 24 | 13.3 | 58.5 | 19.8 | 7.3 | 6.6 | 14 | 72.2 |
| **State of Palestine** | 1.4 | 0.1 | 1.5 | 96.9 | 1.6 | 0 | 58.8 | 7 | 34.1 |
| **Suriname** | 3.6 | 5.4 | 9.5 | 81.5 | 11.6 | 1.4 | 96 | 2 | 0.6 |
| **Tanzania** | 23.4 | 31 | 20.5 | 25.1 | 31.1 | 39.7 | 16.8 | 23.6 | 19.9 |
| **The Gambia** | 1.1 | 40.8 | 15.1 | 42.9 | 27.5 | 11.3 | 45.5 | 42.6 | 0.5 |
| **Timor-Leste** | 30.4 | 7 | 10.2 | 52.4 | 15.4 | 18.3 | 6.5 | 14 | 61.2 |
| **Tunisia** | 1.5 | 0.1 | 2.3 | 96.1 | 2.4 | 1.4 | 86.4 | 8.8 | 3.4 |
| **Uganda** | 7.6 | 58.7 | 17.2 | 16.5 | 41.2 | 20.7 | 34.3 | 32 | 13 |
| **Zambia** | 12.6 | 37.4 | 22.1 | 27.9 | 40.8 | 31.4 | 10.7 | 33.7 | 24.2 |
| **Zimbabwe** | 24.9 | 10.1 | 37.3 | 27.8 | 54.7 | 23.7 | 41 | 34.8 | 0.5 |
| **Overall** | **29.3** | **12.9** | **13** | **44.8** | **24.1** | **13.6** | **17.8** | **24.7** | **43.9** |

*^1^ A reference category for regression analysis*

*^2^ Not shared is the reference category for whether or not the sanitation facility is shared*

**Figure S1.** Map of prevalence (%) of DIL across (A) North Africa and the middle East and Sub-Saharan Africa with an inset for Sao Tome and Principe, (B) South Asia and Southeast Asia and the Pacific with an inset for the Maldives, (C) Latin America and the Caribbean.

**
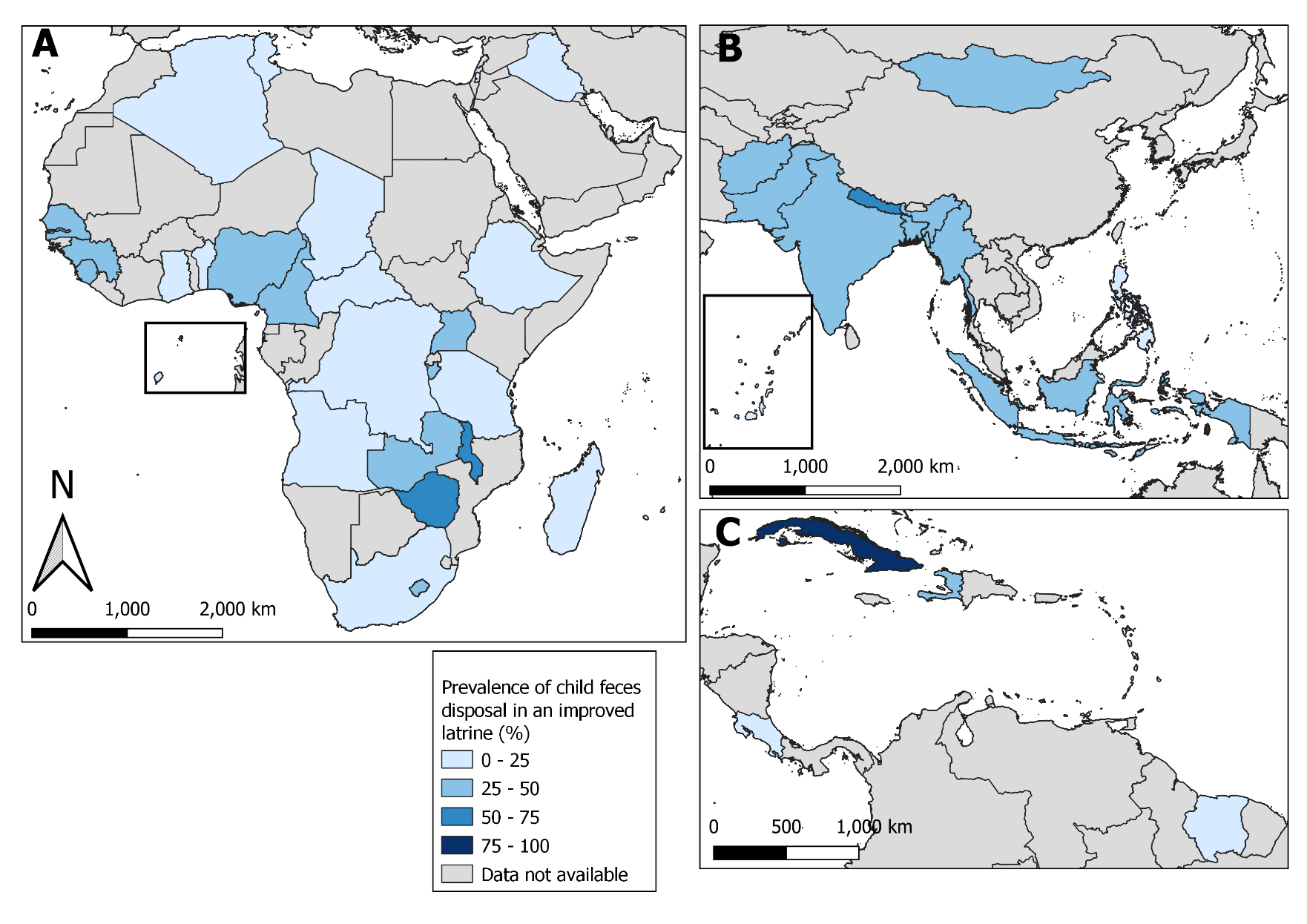
**

**Figure S2.** Prevalence of direct improved latrine use among child’s age categories across countries for which data on at least 0 to 2-year-olds was collected. Prevalence of direct latrine use of any kind is shown in the main text Fig. 2. Prevalence values are shown using denormalized weights.


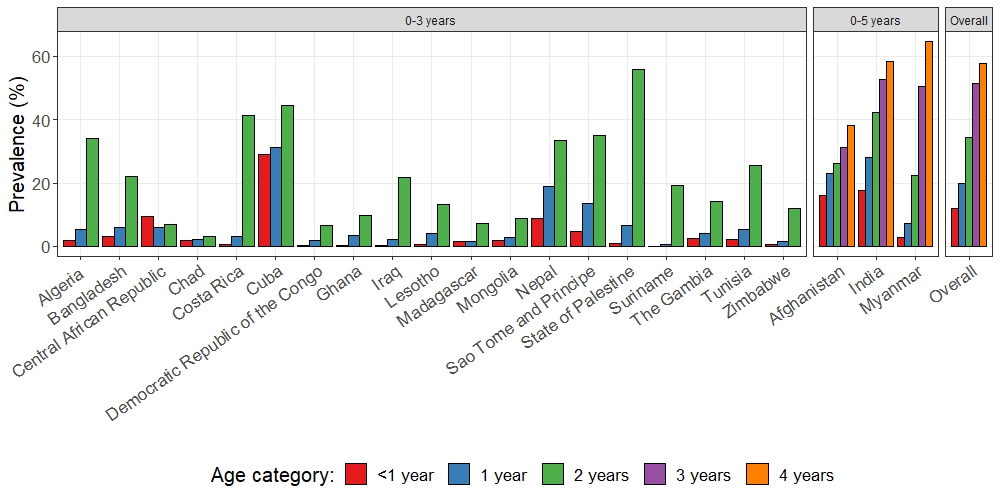


**Table S4.** Prevalence of direct toilet or latrine use by age for countries with data on children 0-2 or 0-4 years old. Percentages are shown using denormalized weights, and represent those using any latrine amongst households with access to any latrine, or those using a safe or basic latrine amongst households with access to a safe or basic latrine, respectively.

|  |  | **Any latrine** | **Improved latrine** |
| --- | --- | --- | --- |
| **Country** | **Age** | **Yes (%)** | **Yes (%)** |
| **Afghanistan** | **0** | **16.3** | **16** |
| **Afghanistan** | **1** | **22.3** | **22.9** |
| **Afghanistan** | **2** | **24.5** | **26.2** |
| **Afghanistan** | **3** | **33.7** | **31.2** |
| **Afghanistan** | **4** | **40.7** | **38.3** |
| **Afghanistan** | **Total** | **23.8** | **23.8** |
| **Algeria** | **0** | **2** | **2** |
| **Algeria** | **1** | **5.2** | **5.2** |
| **Algeria** | **2** | **34** | **34.2** |
| **Algeria** | **Total** | **13.9** | **13.8** |
| **Bangladesh** | **0** | **2.7** | **3.1** |
| **Bangladesh** | **1** | **5** | **6** |
| **Bangladesh** | **2** | **19.7** | **22.1** |
| **Bangladesh** | **Total** | **9.2** | **10.4** |
| **Central African Republic** | **0** | **3.3** | **9.4** |
| **Central African Republic** | **1** | **4.4** | **5.9** |
| **Central African Republic** | **2** | **6.5** | **6.8** |
| **Central African Republic** | **Total** | **4.7** | **7.4** |
| **Chad** | **0** | **1.8** | **2** |
| **Chad** | **1** | **1.6** | **2.3** |
| **Chad** | **2** | **2.8** | **3.2** |
| **Chad** | **Total** | **2** | **2.5** |
| **Costa Rica** | **0** | **0.4** | **0.5** |
| **Costa Rica** | **1** | **3** | **3.2** |
| **Costa Rica** | **2** | **43** | **41.4** |
| **Costa Rica** | **Total** | **16.1** | **15.7** |
| **Cuba** | **0** | **26.1** | **29.1** |
| **Cuba** | **1** | **28.4** | **31.2** |
| **Cuba** | **2** | **40.9** | **44.4** |
| **Cuba** | **Total** | **31.3** | **34.5** |
| **Democratic Republic of the Congo** | **0** | **1.1** | **0.2** |
| **Democratic Republic of the Congo** | **1** | **1.4** | **2** |
| **Democratic Republic of the Congo** | **2** | **4** | **6.5** |
| **Democratic Republic of the Congo** | **Total** | **2.1** | **2.7** |
| **Ghana** | **0** | **0.2** | **0.2** |
| **Ghana** | **1** | **1.8** | **3.4** |
| **Ghana** | **2** | **7.2** | **9.8** |
| **Ghana** | **Total** | **3** | **4.4** |
| **India** | **0** | **14.5** | **17.6** |
| **India** | **1** | **25.2** | **28.1** |
| **India** | **2** | **38.9** | **42.2** |
| **India** | **3** | **50** | **52.6** |
| **India** | **4** | **55.5** | **58.4** |
| **India** | **Total** | **33.7** | **37.6** |
| **Iraq** | **0** | **0.4** | **0.3** |
| **Iraq** | **1** | **2.5** | **2.3** |
| **Iraq** | **2** | **22.1** | **21.7** |
| **Iraq** | **Total** | **8.3** | **8.1** |
| **Lesotho** | **0** | **1.3** | **0.5** |
| **Lesotho** | **1** | **3.6** | **4.2** |
| **Lesotho** | **2** | **14.2** | **13.1** |
| **Lesotho** | **Total** | **6.2** | **5.7** |
| **Madagascar** | **0** | **0.9** | **1.7** |
| **Madagascar** | **1** | **1.9** | **1.5** |
| **Madagascar** | **2** | **7.5** | **7.2** |
| **Madagascar** | **Total** | **3.2** | **3** |
| **Mongolia** | **0** | **1.4** | **1.8** |
| **Mongolia** | **1** | **2.5** | **2.8** |
| **Mongolia** | **2** | **7.2** | **8.8** |
| **Mongolia** | **Total** | **3.7** | **4.6** |
| **Myanmar** | **0** | **3.2** | **2.9** |
| **Myanmar** | **1** | **5.9** | **7.4** |
| **Myanmar** | **2** | **22.2** | **22.3** |
| **Myanmar** | **3** | **50.3** | **50.4** |
| **Myanmar** | **4** | **65** | **64.6** |
| **Myanmar** | **Total** | **25.9** | **26** |
| **Nepal** | **0** | **9.3** | **8.8** |
| **Nepal** | **1** | **19.4** | **18.8** |
| **Nepal** | **2** | **34.7** | **33.4** |
| **Nepal** | **Total** | **20.9** | **20** |
| **Sao Tome and Principe** | **0** | **4.8** | **4.8** |
| **Sao Tome and Principe** | **1** | **12.1** | **13.6** |
| **Sao Tome and Principe** | **2** | **34.6** | **35.1** |
| **Sao Tome and Principe** | **Total** | **17.3** | **18** |
| **State of Palestine** | **0** | **1** | **1** |
| **State of Palestine** | **1** | **6.6** | **6.6** |
| **State of Palestine** | **2** | **55.5** | **55.8** |
| **State of Palestine** | **Total** | **20.9** | **21** |
| **Suriname** | **0** | **0** | **0** |
| **Suriname** | **1** | **0.7** | **0.7** |
| **Suriname** | **2** | **19** | **19.4** |
| **Suriname** | **Total** | **7.1** | **7.2** |
| **The Gambia** | **0** | **1.9** | **2.5** |
| **The Gambia** | **1** | **2.8** | **4.1** |
| **The Gambia** | **2** | **10.7** | **14.1** |
| **The Gambia** | **Total** | **5.1** | **7** |
| **Tunisia** | **0** | **2.2** | **2.3** |
| **Tunisia** | **1** | **5.2** | **5.2** |
| **Tunisia** | **2** | **25.4** | **25.5** |
| **Tunisia** | **Total** | **11.1** | **11.2** |
| **Zimbabwe** | **0** | **1.3** | **0.7** |
| **Zimbabwe** | **1** | **2.4** | **1.7** |
| **Zimbabwe** | **2** | **11.5** | **12.1** |
| **Zimbabwe** | **Total** | **5.1** | **5.2** |
| **Overall** | **0** | **9.4** | **12.1** |
| **Overall** | **1** | **16.1** | **20.0** |
| **Overall** | **2** | **28.1** | **34.4** |
| **Overall** | **3** | **49.1** | **51.3** |
| **Overall** | **4** | **55.5** | **57.9** |
| **Overall** | **Total** | **24.5** | **29.4** |

**Table S5**. Proportion of overall dataset with access to any toilet or latrine and with access to a safely managed or basic latrine, broken down by age. These numbers form the denominators for the direct toilet use table and figures above and in the main text.

|  | **Any toilet use** | | **Improved toilet use** | |
| --- | --- | --- | --- | --- |
| **Age** | **N** | **Yes (%)** | **N** | **Yes (%)** |
| 0 | 21,710,178 | 65.5 | 14,141,062 | 42.7 |
| 1 | 22,043,049 | 65.7 | 14,465,693 | 43.1 |
| 2 | 19,772,736 | 68.8 | 12,920,673 | 44.9 |
| 3 | 9,090,710 | 61.1 | 6,983,829 | 46.9 |
| 4 | 7,498,186 | 60.9 | 5,828,573 | 47.4 |
| **Total** | **80,114,860** | **65.3** | **54,339,830** | **44.3** |

**Table S6.** Full Poisson Regression table for DAL (Analysis 1)

| **Variable** | **Levels** | **aPR** | **95% CI** | ***p*** |
| --- | --- | --- | --- | --- |
| Child Sex (Female) | Male | 0.99 | (0.98 - 1.001) | 0.075 |
| Urbanicity (Rural) | Urban | 1.11 | (1.083 - 1.13) | <0.001 |
| Breastfeeding (Not breastfeeding) | Yes | 0.95 | (0.93 - 0.96) | <0.001 |
| Mother's Education (Less than primary) | Primary | 1.02 | (0.99 - 1.05) | 0.13 |
|  | Secondary or higher | 0.99 | (0.97 - 1.02) | 0.528 |
| Number of children under 5 (1) | 2 or more | 1.03 | (1.01 - 1.05) | <0.001 |
| Number of persons in the household (<5) | 6 or more | 0.99 | (0.97 - 1) | 0.054 |
| JMP Water Ladder (Surface water) | Limited | 0.95 | (0.91 - 0.99) | 0.008 |
|  | Basic | 0.97 | (0.94 - 1.01) | 0.14 |
|  | Safely Managed | 0.99 | (0.98 - 1.05) | 0.384 |
| JMP Sanitation Ladder (Open defecation) | Unimproved | 1.18 | (1.09 - 1.28) | <0.001 |
|  | Limited | 1.15 | (1.06 - 1.25) | 0.001 |
|  | Basic/ Safely Managed | 1.18 | (1.1 - 1.27) | <0.001 |
| Wealth quintiles (Poorest 20%) | Poorer | 1.08 | (1.05 - 1.11) | <0.001 |
|  | Middle | 1.19 | (1.16 - 1.23) | <0.001 |
|  | Richer | 1.31 | (1.27 - 1.35) | <0.001 |
|  | Richest | 1.41 | (1.36 - 1.46) | <0.001 |
| Child Age (Years) | | 1.19 | (1.18 - 1.2) | <0.001 |
| Mother Age (years) | | 1.001 | (1 - 1.002) | 0.046 |
| Shared Toilet (Not shared) | Shared | 0.95 | (0.91 - 0.98) | 0.006 |
|  |  |  |  |  |
| Country | Burundi | 1.150 | (1.1 - 1.21) | 0.006 |
|  | CAR | 0.809 | (0.76 - 0.86) | <0.001 |
|  | Chad | 0.499 | (0.45 - 0.55) | <0.001 |
|  | DRC | 0.903 | (0.84 - 0.97) | <0.001 |
|  | Ghana | 0.393 | (0.36 - 0.43) | 0.003 |
|  | Guinea | 0.919 | (0.86 - 0.99) | <0.001 |
|  | Madagascar | 0.627 | (0.58 - 0.67) | 0.019 |
|  | Malawi | 1.347 | (1.28 - 1.41) | <0.001 |
|  | Sao Tome Principe | 0.403 | (0.34 - 0.48) | <0.001 |
|  | Senegal | 0.960 | (0.89 - 1.04) | <0.001 |
|  | Sierra Leone | 1.046 | (0.99 - 1.11) | 0.303 |
|  | South Africa | 0.219 | (0.17 - 0.28) | 0.139 |
|  | Tanzania | 1.099 | (1.04 - 1.16) | <0.001 |
|  | Gambia | 1.061 | (1 - 1.12) | 0.001 |
|  | Zambia | 1.210 | (1.15 - 1.27) | 0.038 |
|  | Zimbabwe | 1.148 | (1.09 - 1.21) | <0.001 |
|  | Nepal | 1.037 | (0.98 - 1.1) | <0.001 |
|  | Uganda | 1.234 | (1.18 - 1.29) | 0.227 |
|  | Nigeria | 0.983 | (0.93 - 1.04) | <0.001 |
|  | Ethiopia | 0.761 | (0.7 - 0.83) | 0.528 |
|  | Lesotho | 0.900 | (0.84 - 0.97) | <0.001 |
|  | Iraq | 0.208 | (0.18 - 0.24) | 0.004 |
|  | Palestine | 0.323 | (0.3 - 0.35) | <0.001 |
|  | Tunisia | 0.202 | (0.18 - 0.23) | <0.001 |
|  | Bangladesh | 0.725 | (0.69 - 0.77) | <0.001 |
|  | Indonesia | 0.657 | (0.62 - 0.7) | <0.001 |
|  | Mongolia | 0.709 | (0.66 - 0.76) | <0.001 |
|  | Myanmar | 0.736 | (0.7 - 0.78) | <0.001 |
|  | Philippines | 0.156 | (0.13 - 0.18) | <0.001 |
|  | Timor-Leste | 0.407 | (0.36 - 0.46) | <0.001 |
|  | Costa Rica | 0.279 | (0.24 - 0.33) | <0.001 |
|  | Cuba | 1.200 | (1.13 - 1.28) | <0.001 |
|  | Haiti | 1.209 | (1.14 - 1.28) | <0.001 |
|  | Suriname | 0.173 | (0.15 - 0.2) | <0.001 |
|  | Algeria | 0.238 | (0.22 - 0.26) | <0.001 |
|  | Afghanistan | 0.450 | (0.4 - 0.51) | <0.001 |
|  | India | 0.590 | (0.56 - 0.62) | <0.001 |
|  | Maldives | 0.121 | (0.08 - 0.18) | <0.001 |
|  | Pakistan | 0.572 | (0.52 - 0.63) | <0.001 |
|  | Angola | 0.521 | (0.47 - 0.58) | <0.001 |
|  | Benin | 0.876 | (0.82 - 0.93) | <0.001 |
|  | Cameroon | 1.000 | (0 - 0) |  |
|  | _cons | 0.431 | (0.39 - 0.48) | 0 |

**Table S7.** Full Poisson Regression table for DIL (Analysis 2)

| **Variable** | **Levels** | **aPR** | **95% CI** | **p** |
| --- | --- | --- | --- | --- |
| Child Sex (Female) | Male | 0.997 | (0.98 - 1.014) | 0.748 |
| Child Age (Years) | | 1.20 | (1.19 - 1.22) | <0.001 |
| Mother Age (years) | | 1.004 | (1.003 - 1.006) | <0.001 |
| Urbanicity (Rural) | Urban | 1.23 | (1.2 - 1.27) | <0.001 |
| Breastfeeding (Not breastfeeding) | Yes | 0.94 | (0.92 - 0.96) | <0.001 |
| Mother's Education (Less than primary) | Primary | 1.17 | (1.12 - 1.209) | <0.001 |
|  | Secondary or higher | 1.18 | (1.14 - 1.23) | <0.001 |
| Number of children under 5 (1) | 2 or more | 1.03 | (1.01 - 1.05) | 0.003 |
| Number of persons in the household (<5) | 6 or more | 0.96 | (0.94 - 0.98) | <0.001 |
| JMP Water Ladder (Surface water) | Limited | 0.86 | (0.8 - 0.92) | <0.001 |
|  | Basic | 1.09 | (1.02 - 1.15) | 0.006 |
|  | Safely Managed | 1.38 | (1.3 - 1.46) | <0.001 |
| Wealth quintiles (Poorest) | Poorer | 2.12 | (2.01 - 2.24) | <0.001 |
|  | Middle | 3.29 | (3.12 - 3.46) | <0.001 |
|  | Richer | 4.59 | (4.36 - 4.84) | <0.001 |
|  | Richest | 5.48 | (5.19 - 5.78) | <0.001 |
|  |  |  |  |  |
| Country | Burundi | 1.186 | (1.08 - 1.3) | <0.001 |
|  | CAR | 0.359 | (0.31 - 0.42) | <0.001 |
|  | Chad | 0.219 | (0.19 - 0.26) | <0.001 |
|  | DRC | 0.595 | (0.51 - 0.7) | <0.001 |
|  | Ghana | 0.429 | (0.37 - 0.49) | <0.001 |
|  | Guinea | 0.927 | (0.83 - 1.04) | 0.183 |
|  | Madagascar | 0.209 | (0.18 - 0.25) | <0.001 |
|  | Malawi | 2.179 | (1.99 - 2.38) | <0.001 |
|  | SaoTomePrincipe | 0.380 | (0.31 - 0.47) | <0.001 |
|  | Senegal | 1.211 | (1.06 - 1.38) | 0.004 |
|  | SierraLeone | 1.070 | (0.96 - 1.19) | 0.205 |
|  | SouthAfrica | 0.215 | (0.15 - 0.3) | <0.001 |
|  | Tanzania | 0.804 | (0.73 - 0.89) | <0.001 |
|  | Gambia | 1.272 | (1.15 - 1.41) | <0.001 |
|  | Zambia | 1.076 | (0.97 - 1.19) | 0.159 |
|  | Zimbabwe | 1.496 | (1.37 - 1.64) | <0.001 |
|  | Nepal | 2.101 | (1.91 - 2.31) | <0.001 |
|  | Uganda | 0.810 | (0.74 - 0.89) | <0.001 |
|  | Nigeria | 0.879 | (0.8 - 0.96) | 0.006 |
|  | Ethiopia | 0.173 | (0.14 - 0.21) | <0.001 |
|  | Lesotho | 1.262 | (1.13 - 1.41) | <0.001 |
|  | Iraq | 0.393 | (0.34 - 0.46) | <0.001 |
|  | Palestine | 0.609 | (0.54 - 0.68) | <0.001 |
|  | Tunisia | 0.403 | (0.35 - 0.47) | <0.001 |
|  | Bangladesh | 1.343 | (1.23 - 1.47) | <0.001 |
|  | Indonesia | 0.799 | (0.73 - 0.87) | <0.001 |
|  | Mongolia | 1.148 | (1.04 - 1.27) | 0.007 |
|  | Myanmar | 0.684 | (0.62 - 0.76) | <0.001 |
|  | Philippines | 0.210 | (0.18 - 0.25) | <0.001 |
|  | TimorLeste | 0.442 | (0.38 - 0.51) | <0.001 |
|  | CostaRica | 0.595 | (0.49 - 0.73) | <0.001 |
|  | Cuba | 2.126 | (1.91 - 2.37) | <0.001 |
|  | Haiti | 1.134 | (1.02 - 1.26) | 0.022 |
|  | Suriname | 0.369 | (0.31 - 0.44) | <0.001 |
|  | Algeria | 0.464 | (0.41 - 0.52) | <0.001 |
|  | Afghanistan | 0.681 | (0.59 - 0.79) | <0.001 |
|  | India | 0.538 | (0.5 - 0.58) | <0.001 |
|  | Maldives | 0.171 | (0.12 - 0.25) | <0.001 |
|  | Pakistan | 0.781 | (0.69 - 0.88) | <0.001 |
|  | Angola | 0.581 | (0.51 - 0.66) | <0.001 |
|  | Benin | 0.511 | (0.45 - 0.58) | <0.001 |
|  | Cameroon | 1.000 | (0 - 0) |  |
|  | _cons | 0.070 | (0.06 - 0.08) | <0.001 |

**Table S8.** Poisson regression for DAL after restricting the dataset to just those with household access to a latrine (Analysis 3), and for DIL after restricting the dataset to just those with household access to an improved latrine (Analysis 4).

|  |  | **DAL** | | | **DIL** | | |
| --- | --- | --- | --- | --- | --- | --- | --- |
| **Variable** | **Level(s)** | **aPR** | **95% CI** | ***p-*value** | **aPR** | **95% CI** | ***p*-value** |
| **Child Sex (Female)** | **Male** | 0.99 | (0.98 - 1) | 0.073 | 0.99 | (0.98 - 1.01) | 0.324 |
| **Urbanicity (Rural)** | **Urban** | 1.10 | (1.08 - 1.13) | <0.001 | 1.10 | (1.07 - 1.12) | <0.001 |
| **Child Age (years)** | | 1.19 | (1.18 - 1.2) | <0.001 | 1.18 | (1.17 - 1.19) | <0.001 |
| **Mother age (years)** | | 1.00 | (1 - 1) | 0.06 | 1.00 | (1 - 1) | 0.003 |
| **Breastfeeding (Not breastfeeding)** | **Yes** | 0.95 | (0.93 - 0.96) | <0.001 | 0.93 | (0.92 - 0.95) | <0.001 |
| **Mother Education (Less than primary)** | **Primary** | 1.02 | (1 - 1.05) | 0.078 | 1.04 | (1.01 - 1.08) | 0.008 |
|  | **Secondary or higher** | 0.99 | (0.97 - 1.02) | 0.485 | 1.01 | (0.98 - 1.04) | 0.445 |
| **Number of children under 5 (1)** | **2 or more** | 1.03 | (1.01 - 1.04) | <0.001 | 1.03 | (1.01 - 1.05) | 0.001 |
| **Number of persons in the household (<5)** | **6 or more** | 0.99 | (0.97 - 1) | 0.044 | 0.99 | (0.98 - 1.01) | 0.297 |
| **JMP Water Ladder (Surface water and unimproved)** | **Limited** | 0.94 | (0.9 - 0.98) | 0.005 | 0.88 | (0.84 - 0.92) | <0.001 |
|  | **Basic** | 0.97 | (0.93 - 1) | 0.075 | 0.92 | (0.88 - 0.96) | <0.001 |
|  | **Safely Managed** | 1.01 | (0.97 - 1.04) | 0.699 | 0.97 | (0.94 - 1.01) | 0.188 |
| **JMP Sanitation Ladder (Unimproved)** | **Limited** | 1.00 | (0.96 - 1.04) | 0.927 |  |  |  |
|  | **Basic/ Safely Managed** | 1.00 | (0.97 - 1.03) | 0.797 |  |  |  |
| **Wealth quintile (Poorest 20%)** | **Poorer** | 1.06 | (1.03 - 1.09) | <0.001 | 1.13 | (1.08 - 1.18) | <0.001 |
|  | **Middle** | 1.17 | (1.13 - 1.21) | <0.001 | 1.26 | (1.21 - 1.31) | <0.001 |
|  | **Richer** | 1.28 | (1.24 - 1.32) | <0.001 | 1.39 | (1.33 - 1.45) | <0.001 |
|  | **Richest** | 1.39 | (1.34 - 1.44) | <0.001 | 1.50 | (1.44 - 1.57) | <0.001 |
| **Shared Toilet (Not shared)** | **Shared** | 0.93 | (0.89 - 0.96) | <0.001 |  |  |  |
|  |  |  |  |  |  |  |  |
| **Country** | **Burundi** | 1.15 | (1.09 - 1.2) | <0.001 | 1.14 | (1.08 - 1.21) | <0.001 |
|  | **CAR** | 0.81 | (0.76 - 0.87) | <0.001 | 0.85 | (0.78 - 0.93) | <0.001 |
|  | **Chad** | 0.52 | (0.48 - 0.58) | <0.001 | 0.63 | (0.57 - 0.7) | <0.001 |
|  | **DRC** | 0.91 | (0.85 - 0.97) | 0.004 | 0.93 | (0.86 - 1) | 0.048 |
|  | **Ghana** | 0.39 | (0.36 - 0.44) | <0.001 | 0.38 | (0.34 - 0.42) | <0.001 |
|  | **Guinea** | 0.92 | (0.86 - 0.99) | 0.023 | 0.93 | (0.86 - 1) | 0.052 |
|  | **Madagascar** | 0.63 | (0.59 - 0.68) | <0.001 | 0.67 | (0.6 - 0.75) | <0.001 |
|  | **Malawi** | 1.34 | (1.28 - 1.41) | <0.001 | 1.36 | (1.28 - 1.43) | <0.001 |
|  | **Sao Tome and Principe** | 0.41 | (0.35 - 0.48) | <0.001 | 0.43 | (0.37 - 0.51) | <0.001 |
|  | **Senegal** | 0.96 | (0.89 - 1.04) | 0.36 | 0.94 | (0.86 - 1.03) | 0.186 |
|  | **Sierra Leone** | 1.05 | (0.99 - 1.11) | 0.119 | 1.00 | (0.94 - 1.07) | 0.936 |
|  | **South Africa** | 0.22 | (0.17 - 0.28) | <0.001 | 0.20 | (0.15 - 0.27) | <0.001 |
|  | **Tanzania** | 1.10 | (1.04 - 1.16) | 0.001 | 1.06 | (1 - 1.13) | 0.045 |
|  | **Gambia** | 1.06 | (1 - 1.12) | 0.04 | 1.00 | (0.94 - 1.07) | 0.945 |
|  | **Zambia** | 1.21 | (1.15 - 1.27) | <0.001 | 1.15 | (1.08 - 1.22) | <0.001 |
|  | **Zimbabwe** | 1.15 | (1.09 - 1.21) | <0.001 | 1.12 | (1.06 - 1.19) | <0.001 |
|  | **Nepal** | 1.03 | (0.97 - 1.1) | 0.278 | 1.10 | (1.03 - 1.17) | 0.004 |
|  | **Uganda** | 1.23 | (1.18 - 1.29) | <0.001 | 1.14 | (1.08 - 1.21) | <0.001 |
|  | **Nigeria** | 0.98 | (0.93 - 1.03) | 0.423 | 0.89 | (0.83 - 0.94) | <0.001 |
|  | **Ethiopia** | 0.76 | (0.7 - 0.83) | <0.001 | 0.69 | (0.6 - 0.79) | <0.001 |
|  | **Lesotho** | 0.90 | (0.84 - 0.97) | 0.004 | 0.90 | (0.83 - 0.98) | 0.011 |
|  | **Iraq** | 0.20 | (0.18 - 0.23) | <0.001 | 0.21 | (0.18 - 0.24) | <0.001 |
|  | **Palestine** | 0.32 | (0.29 - 0.35) | <0.001 | 0.34 | (0.31 - 0.37) | <0.001 |
|  | **Tunisia** | 0.20 | (0.18 - 0.23) | <0.001 | 0.21 | (0.19 - 0.25) | <0.001 |
|  | **Bangladesh** | 0.72 | (0.68 - 0.76) | <0.001 | 0.78 | (0.73 - 0.83) | <0.001 |
|  | **Indonesia** | 0.68 | (0.64 - 0.72) | <0.001 | 0.69 | (0.65 - 0.73) | <0.001 |
|  | **Mongolia** | 0.71 | (0.66 - 0.76) | <0.001 | 0.73 | (0.67 - 0.78) | <0.001 |
|  | **Myanmar** | 0.73 | (0.7 - 0.78) | <0.001 | 0.73 | (0.68 - 0.78) | <0.001 |
|  | **Philippines** | 0.16 | (0.14 - 0.18) | <0.001 | 0.15 | (0.13 - 0.18) | <0.001 |
|  | **Timor-Leste** | 0.42 | (0.37 - 0.47) | <0.001 | 0.43 | (0.38 - 0.49) | <0.001 |
|  | **Costa Rica** | 0.28 | (0.24 - 0.32) | <0.001 | 0.30 | (0.25 - 0.35) | <0.001 |
|  | **Cuba** | 1.19 | (1.12 - 1.27) | <0.001 | 1.28 | (1.2 - 1.37) | <0.001 |
|  | **Haiti** | 1.21 | (1.14 - 1.28) | <0.001 | 1.17 | (1.09 - 1.25) | <0.001 |
|  | **Suriname** | 0.17 | (0.15 - 0.2) | <0.001 | 0.18 | (0.16 - 0.22) | <0.001 |
|  | **Algeria** | 0.24 | (0.21 - 0.26) | <0.001 | 0.25 | (0.22 - 0.27) | <0.001 |
|  | **Afghanistan** | 0.45 | (0.4 - 0.51) | <0.001 | 0.48 | (0.42 - 0.54) | <0.001 |
|  | **India** | 0.59 | (0.56 - 0.62) | <0.001 | 0.61 | (0.58 - 0.64) | <0.001 |
|  | **Maldives** | 0.12 | (0.08 - 0.18) | <0.001 | 0.12 | (0.08 - 0.18) | <0.001 |
|  | **Pakistan** | 0.57 | (0.52 - 0.63) | <0.001 | 0.60 | (0.55 - 0.67) | <0.001 |
|  | **Angola** | 0.52 | (0.47 - 0.58) | <0.001 | 0.50 | (0.45 - 0.56) | <0.001 |
|  | **Benin** | 0.88 | (0.83 - 0.94) | <0.001 | 0.89 | (0.83 - 0.95) | 0.001 |
|  | **Cameroon** | 1.00 | (0 - 0) |  | 1.00 | (0 - 0) |  |
|  | **_cons** | 0.52 | (0.49 - 0.56) | <0.001 | 0.48 | (0.44 - 0.52) | <0.001 |
|  |  |  |  |  |  |  |  |

**Table S9.** Sensitivity analyses results for DAL

|  |  | **Denormalized weights** | | **Equal Country** | | **No India** | | **Children under 2 years** | | **Double Clustering** | | **Youngest under 2 years** | |
| --- | --- | --- | --- | --- | --- | --- | --- | --- | --- | --- | --- | --- | --- |
| **Variable** | **Levels** | **aPR** | **95% CI** | **aPR** | **95% CI** | **aPR** | **95% CI** | **aPR** | **95% CI** | **aPR** | **95% CI** | **aPR** | **95% CI** |
| **Child Sex (Female)** | Male | 0.99 | (0.98 - 1.001) | 0.99 | (0.98 - 1.004) | 0.99 | (0.97 - 1) | 0.99 | (0.97 - 1.01) | 0.99 | (0.98 - 1.001) | 0.99 | (0.98 - 1.002) |
| **Urbanicity (Rural)** | Urban | 1.11 | (1.083 - 1.13) | 1.29 | (1.28 - 1.309) | 1.07 | (1.038 - 1.11) | 1.10 | (1.07 - 1.14) | 1.11 | (1.08 - 1.13) | 1.11 | (1.088 - 1.14) |
| **Breastfeeding (Not breastfeeding)** | Yes | 0.95 | (0.93 - 0.96) | 1.001 | (1.0005 - 1.003) | 0.993 | (0.97 - 1.02) | 1.01 | (0.99 - 1.04) | 0.95 | (0.93 - 0.96) | 0.95 | (0.93 - 0.97) |
| **Mother's Education (Less than primary)** | Primary | 1.02 | (0.99 - 1.05) | 1.02 | (1 - 1.05) | 1.00 | (0.97 - 1.04) | 1.01 | (0.98 - 1.05) | 1.02 | (0.99 - 1.05) | 1.02 | (0.99 - 1.05) |
|  | Secondary or higher | 0.99 | (0.97 - 1.02) | 0.99 | (0.97 - 1) | 0.95 | (0.92 - 0.99) | 0.96 | (0.93 - 1) | 0.99 | (0.97 - 1.02) | 0.99 | (0.96 - 1.02) |
| **Number of children under 5 (1)** | 2 or more | 1.03 | (1.01 - 1.05) | 1.04 | (1.02 - 1.059) | 1.02 | (1 - 1.05) | 1.04 | (1.01 - 1.06) | 1.03 | (1.01 - 1.05) | 1.03 | (1.01 - 1.04) |
| **Number of persons in the household (<5)** | 6 or more | 0.99 | (0.97 - 1) | 1.04 | (1.01 - 1.06) | 0.99 | (0.97 - 1.02) | 0.99 | (0.97 - 1.01) | 0.99 | (0.97 - 1) | 0.99 | (0.97 - 1.002) |
| **JMP Water Ladder (Surface water)** | Limited | 0.95 | (0.91 - 0.99) | 1.02 | (1.01 - 1.04) | 1.02 | (0.97 - 1.06) | 0.95 | (0.91 - 1) | 0.95 | (0.91 - 0.99) | 0.92 | (0.89 - 0.96) |
|  | Basic | 0.97 | (0.94 - 1.01) | 0.99 | (0.97 - 1) | 1.04 | (1 - 1.09) | 0.99 | (0.95 - 1.02) | 0.97 | (0.94 - 1.01) | 0.95 | (0.92 - 0.99) |
|  | Safely Managed | 0.99 | (0.98 - 1.05) | 1.03 | (1 - 1.06) | 1.02 | (0.98 - 1.07) | 1.01 | (0.97 - 1.06) | 1.02 | (0.98 - 1.05) | 0.99 | (0.96 - 1.03) |
| **JMP Sanitation Ladder (Open defecation)** | Unimproved | 1.18 | (1.09 - 1.28) | 1.05 | (1.02 - 1.07) | 1.40 | (1.25 - 1.58) | 1.31 | (1.17 - 1.47) | 1.18 | (1.09 - 1.28) | 1.19 | (1.1 - 1.29) |
|  | Limited | 1.15 | (1.06 - 1.25) | 1.05 | (1.02 - 1.08) | 1.42 | (1.26 - 1.61) | 1.24 | (1.1 - 1.41) | 1.15 | (1.06 - 1.25) | 1.15 | (1.06 - 1.26) |
|  | Basic/ Safely Managed | 1.18 | (1.1 - 1.27) | 1.36 | (1.24 - 1.5) | 1.45 | (1.29 - 1.63) | 1.30 | (1.17 - 1.45) | 1.18 | (1.1 - 1.27) | 1.18 | (1.09 - 1.27) |
| **Wealth quintiles (Poorest 20%)** | Poorer | 1.08 | (1.05 - 1.11) | 1.41 | (1.28 - 1.55) | 1.10 | (1.06 - 1.14) | 1.10 | (1.06 - 1.14) | 1.08 | (1.05 - 1.11) | 1.10 | (1.07 - 1.14) |
|  | Middle | 1.19 | (1.16 - 1.23) | 1.39 | (1.27 - 1.53) | 1.18 | (1.14 - 1.22) | 1.20 | (1.16 - 1.25) | 1.19 | (1.16 - 1.23) | 1.22 | (1.18 - 1.26) |
|  | Richer | 1.31 | (1.27 - 1.35) | 1.04 | (1.02 - 1.07) | 1.20 | (1.15 - 1.25) | 1.29 | (1.24 - 1.34) | 1.31 | (1.27 - 1.35) | 1.34 | (1.29 - 1.39) |
|  | Richest | 1.41 | (1.36 - 1.46) | 1.07 | (1.05 - 1.1) | 1.19 | (1.13 - 1.239) | 1.35 | (1.3 - 1.42) | 1.41 | (1.36 - 1.46) | 1.45 | (1.4 - 1.51) |
| **Child Age (Years)** |  | 1.19 | (1.18 - 1.2) | 1.09 | (1.06 - 1.12) | 1.26 | (1.25 - 1.28) | 1.27 | (1.25 - 1.3) | 1.19 | (1.18 - 1.2) | 1.19 | (1.18 - 1.19) |
| **Mother Age (years)** |  | 1.001 | (1 - 1.002) | 1.09 | (1.06 - 1.12) | 1.00 | (0.998 - 1.001) | 1.00 | (0.999 - 1.002) | 1.001 | (1 - 1.002) | 1.00 | (1 - 1.002) |
| **Shared Toilet (Not shared)** | Shared | 0.95 | (0.91 - 0.98) | 0.96 | (0.93 - 0.98) | 0.94 | (0.9 - 0.98) | 0.94 | (0.9 - 0.99) | 0.95 | (0.91 - 0.98) | 0.94 | (0.9 - 0.98) |

**Table S10.** Sensitivity Analyses results for DIL

|  |  | **Denormalized weights** | | **Equal Country** | | **No India** | | **Children under 2 years** | | **Double Clustering** | | **Youngest under 2 years** | |
| --- | --- | --- | --- | --- | --- | --- | --- | --- | --- | --- | --- | --- | --- |
| **Variable** | **Levels** | **aPR** | **95% CI** | **aPR** | **95% CI** | **aPR** | **95% CI** | **aPR** | **95% CI** | **aPR** | **95% CI** | **aPR** | **95% CI** |
| **Child Sex (Female)** | Male | 0.997 | (0.98 - 1.014) | 0.99 | (0.98 - 1.013) | 1.00 | (0.98 - 1.027) | 1.002 | (0.98 - 1.026) | 1.00 | (0.98 - 1.014) | 0.997 | (0.98 - 1.01) |
| **Child Age (Years)** |  | 1.20 | (1.19 - 1.22) | 1.31 | (1.29 - 1.34) | 1.28 | (1.26 - 1.3) | 1.30 | (1.27 - 1.34) | 1.20 | (1.19 - 1.22) | 1.20 | (1.19 - 1.21) |
| **Mother Age (years)** |  | 1.004 | (1.003- 1.006) | 1.004 | (1.002 - 1.005) | 1.001 | (0.999 - 1.003) | 1.003 | (1.001 - 1.005) | 1.004 | (1.003- 1.006) | 1.004 | (1.002- 1.006) |
| **Urbanicity (Rural)** | Urban | 1.23 | (1.2 - 1.27) | 1.18 | (1.15 - 1.22) | 1.16 | (1.11 - 1.22) | 1.21 | (1.16 - 1.26) | 1.23 | (1.2 - 1.27) | 1.23 | (1.2 - 1.27) |
| **Breastfeeding (Not breastfeeding)** | Yes | 0.94 | (0.92 - 0.96) | 0.96 | (0.93 - 0.99) | 0.97 | (0.94 - 1) | 1.01 | (0.97 - 1.04) | 0.94 | (0.92 - 0.96) | 0.94 | (0.92 - 0.96) |
| **Mother's Education (Less than primary)** | Primary | 1.17 | (1.12 - 1.209) | 1.13 | (1.09 - 1.166) | 1.08 | (1.03 - 1.138) | 1.11 | (1.06 - 1.168) | 1.17 | (1.12 - 1.21) | 1.15 | (1.11 - 1.197) |
|  | Secondary or higher | 1.18 | (1.14 - 1.23) | 1.22 | (1.18 - 1.26) | 1.07 | (1.02 - 1.14) | 1.09 | (1.04 - 1.15) | 1.18 | (1.14 - 1.23) | 1.16 | (1.12 - 1.2) |
| **Number of children under 5 (1)** | 2 or more | 1.03 | (1.01 - 1.05) | 1.03 | (1.005 - 1.05) | 1.03 | (0.998 - 1.06) | 1.04 | (1.017 - 1.07) | 1.03 | (1.01 - 1.05) | 1.02 | (1.005 - 1.05) |
| **Number of persons in the household (<5)** | 6 or more | 0.96 | (0.94 - 0.98) | 0.99 | (0.97 - 1.01) | 0.98 | (0.95 - 1.01) | 0.97 | (0.94 - 0.99) | 0.96 | (0.94 - 0.98) | 0.96 | (0.94 - 0.97) |
| **JMP Water Ladder (Surface water)** | Limited | 0.86 | (0.8 - 0.92) | 1.29 | (1.23 - 1.36) | 1.24 | (1.15 - 1.34) | 0.90 | (0.84 - 0.97) | 0.86 | (0.8 - 0.92) | 0.78 | (0.73 - 0.83) |
|  | Basic | 1.09 | (1.02 - 1.15) | 1.36 | (1.3 - 1.42) | 1.36 | (1.26 - 1.46) | 1.12 | (1.05 - 1.2) | 1.09 | (1.02 - 1.15) | 1.04 | (0.98 - 1.1) |
|  | Safely Managed | 1.38 | (1.3 - 1.46) | 1.60 | (1.52 - 1.68) | 1.53 | (1.42 - 1.65) | 1.43 | (1.33 - 1.53) | 1.38 | (1.3 - 1.46) | 1.30 | (1.23 - 1.37) |
| **Wealth quintiles (Poorest)** | Poorer | 2.12 | (2.01 - 2.24) | 1.56 | (1.49 - 1.63) | 1.72 | (1.61 - 1.82) | 2.09 | (1.95 - 2.24) | 2.12 | (2.01 - 2.24) | 2.31 | (2.17 - 2.45) |
|  | Middle | 3.29 | (3.12 - 3.46) | 1.92 | (1.83 - 2) | 2.23 | (2.1 - 2.37) | 3.11 | (2.91 - 3.33) | 3.29 | (3.12 - 3.46) | 3.65 | (3.45 - 3.86) |
|  | Richer | 4.59 | (4.36 - 4.84) | 2.23 | (2.13 - 2.33) | 2.60 | (2.44 - 2.76) | 4.15 | (3.88 - 4.44) | 4.59 | (4.36 - 4.84) | 5.15 | (4.87 - 5.45) |
|  | Richest | 5.48 | (5.19 - 5.78) | 2.40 | (2.28 - 2.53) | 2.93 | (2.75 - 3.13) | 4.92 | (4.59 - 5.28) | 5.48 | (5.19 - 5.78) | 6.10 | (5.76 - 6.47) |

**Figure S3.** Child feces disposal in latrines conditional on household access to latrines. (A) Prevalence of DAL as a function of household access to any type of latrine, and (B) Prevalence of DIL as a function of household access to an improved latrine. The solid line represents 100% usage, and the dashed line represents 50% usage. Individual countries shown in red, overall shown in blue. (C) Prevalence of access to any latrine (red) and improved latrines (blue) by country, and (D) prevalence of DAL (green) and DIL (orange) conditional on access by country.


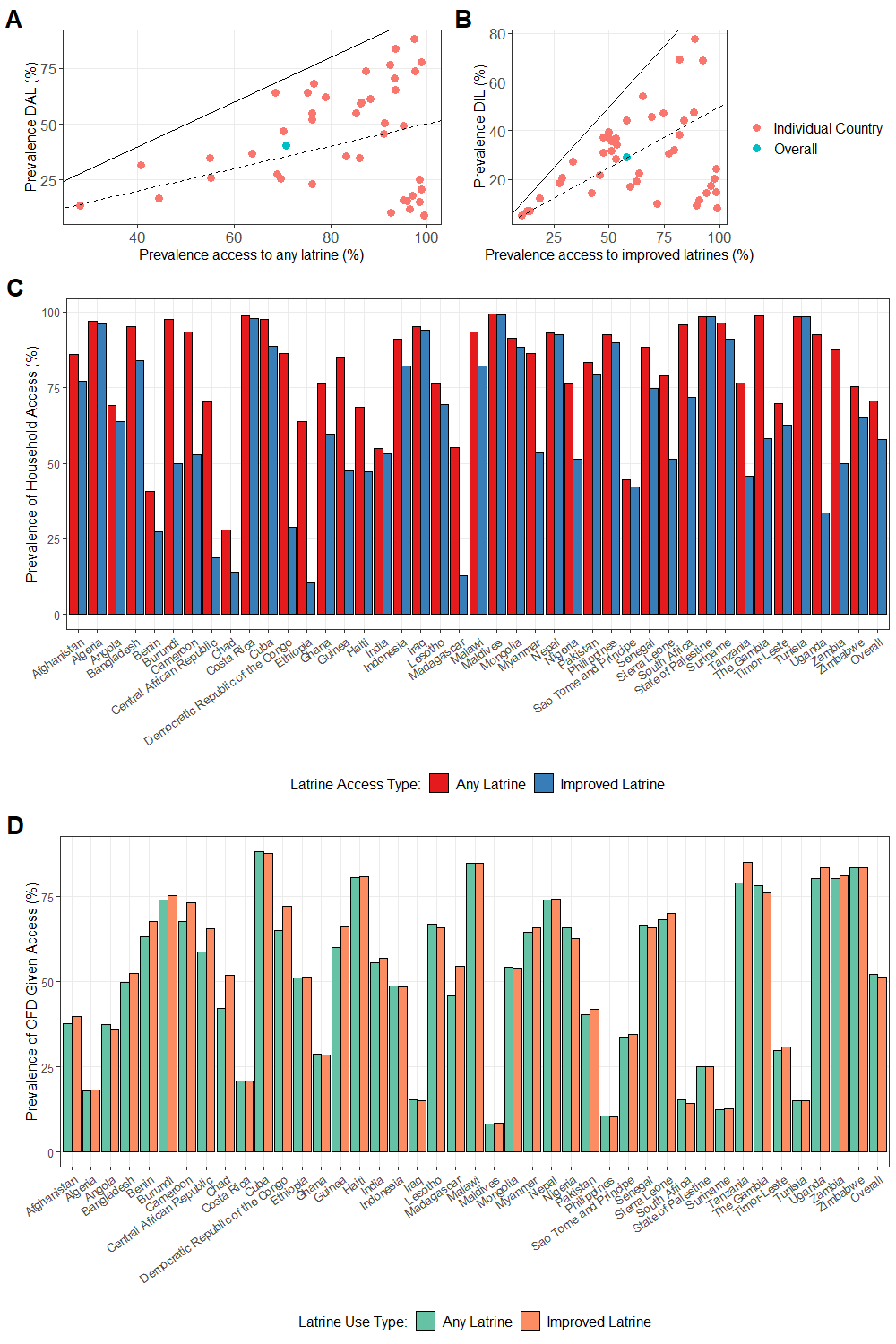

Supplement: Supplementary Tables and Figures [file mmc1.docx]
